# Infantile Hemangioma Originates From A Dysregulated But Not Fully Transformed Multipotent Stem Cell

**DOI:** 10.1038/srep35811

**Published:** 2016-10-27

**Authors:** Shaghayegh Harbi, Rong Wang, Michael Gregory, Nicole Hanson, Keith Kobylarz, Kamilah Ryan, Yan Deng, Peter Lopez, Luis Chiriboga, Paolo Mignatti

**Affiliations:** 1 Department of Medicine, New York University School of Medicine, 550 First Avenue New York, NY 10016, USA; 2 VasculoTox Inc., New York, NY 10001, USA; 3 Department of Neurosurgery, Memorial Sloan Kettering Cancer Center, New York, New York 10065, USA; 4 Department of Pathology, New York University School of Medicine, 550 First Avenue New York, NY 10016, USA; 5 Pfizer Inc., Pearl River, NY 10965, USA; 6 Department of Cell Biology, New York University School of Medicine, 550 First Avenue New York, NY 10016, USA

## Abstract

Infantile hemangioma (IH) is the most common tumor of infancy. Its cellular origin and biological signals for uncontrolled growth are poorly understood, and specific pharmacological treatment is unavailable. To understand the process of hemangioma-genesis we characterized the progenitor hemangioma-derived stem cell (HemSC) and its lineage and non-lineage derivatives. For this purpose we performed a high-throughput (HT) phenotypic and gene expression analysis of HemSCs, and analyzed HemSC-derived tumorspheres. We found that IH is characterized by high expression of genes involved in vasculogenesis, angiogenesis, tumorigenesis and associated signaling pathways. These results show that IH derives from a dysregulated stem cell that remains in an immature, arrested stage of development. The potential biomarkers we identified can afford the development of diagnostic tools and precision-medicine therapies to “rewire” or redirect cellular transitions at an early stage, such as signaling pathways or immune response modifiers.

Hemangiomas are common, benign, vascular neoplasms that occur in 4–12% of infants^1^,^2^,^3^,^4^,^5^,^6^. Termed infantile hemangiomas (IH) and deemed as the most common tumor in infancy, they vary tremendously from small, benign growths to large, function- or even life-threatening tumors^7^,^8^,^9^. IH presents either at birth or during the first year of life, and is characterized by initial rapid growth followed by spontaneous, slow regression. The etiopathogenesis of IH is poorly understood, and the cellular origin and biological signals for uncontrolled growth remain elusive. Virchow (1860) proposed an angioblastic origin, Pack and Miller (1950) described the origin as sequestered embryonic tissue^5^,^10^, while Folkman (1998) described IH as an “angiogenic disease” with evidence of a placental origin (2005)^11^,^12^,^13^. A number of theories have been proposed to explain the origins and pathogenesis of IH: placenta, metastatic, progenitor cell (a hemangioma-derived multipotential stem cell, based on expression of the stem cell marker CD133), extrinsic factor (hypoxic environment), neural crest/pericyte stem cell theory (pericyte-like stem cell tumors derived from neural crest, capable of adipocyte differentiation), and metastatic niche theory^5^. In 2005, a molecular profile analysis showed high similarity between IH and placental transcriptomes, indicating that IH arises from an embryonic or primitive cell^13^. In 2008, however, the hemangioma-derived stem cell (HemSC) was identified as the cellular origin of IH^14^. *In vivo* xenotransplantation studies showed that HemSCs coinjected with Matrigel recapitulate the dysregulated formation of blood vessels typical of IH. This comprises the generation of microvessels expressing glucose transporter-1 (GLUT1), a diagnostic marker of IH^15^, followed by involution through differentiation into adipocytes. Subsequently, serial xenotransplantation studies provided further information of HemSCs as cellular precursors of IH. HemSCs give rise to several cellular lineages^10^, and lineage studies *in vivo* and *in vitro* revealed clonality (ability to self-renew) and multipotency (ability to differentiate into endothelial, adipocyte, and pericyte cell lineages)^6^. Tumorsphere formation studies showed a replication capacity of 30 tumorsphere passages in culture^16^; with cells expressing GLUT1, vascular endothelial growth factor (VEGF), the embryonic stem cell (SC) marker SALL4 (sal-like 4 [Drosophila]), and the stem/progenitor cell markers Kinase Domain Receptor [KDR/VEGFR-2/CD309] and CD133^16^.

CD133, a cell surface membrane glycoprotein encoded by the *PROM1/PROM2* genes^17^,^18^, is a cell surface marker of both normal stem/progenitor cells (including normal endothelial cells) and neoplastic tumor stem cells (such as medulloblastoma, glioblastoma, prostate, and colon cancer)^17^, and is expressed in the human embryo during the early stages of vascular development (4-week embryo)^19^. The normal human vascular compartment consists of multiple stem and progenitor cells^20^. In embryonic blood vessels, stem and progenitor cells contribute to endothelial cells, pericytes, and hemogenic endothelium; in adult blood vessels, stem and progenitor cells, found in an organ-specific vascular niche, contribute to pericyte, endothelial cell, and mesenchymal lineage-specific cells^20^,^21^. The possible vascular lineage models for normal endothelial cells include the hemangioblast, the hemogenic endothelium and the mesoderm-derived angioblast models. Thus, the normal vascular compartment consists of multiple stem and progenitor cells including adventitial, endothelial, hemangioblast, hemogenic and pericyte progenitor cells, mesenchymal stem/progenitor cell, and vascular stem cell.

At all stages of development, IH are heterogeneous (comprising endothelial cells, pericytes, myeloid cells, fibroblasts, and mast cells) and eventually involute into fibrofatty tissue (comprised of fat, fibroblasts and connective tissue) that replaces the vascular tissue^6^. This heterogeneity may result from multiple stem cells heterogeneously dysregulated at varying stages of development, and/or from a multipotent stem cell arrested in development. In proliferating-phase IH, the HemSC was identified as a rare CD133+ subset comprising approximately 1% of the tumor cell population, with vasculogenic potential (*de novo* vessel formation), clonogenic ability to self-renew, multipotential ability to give rise to several cellular lineages with distinct morphologies and gene expression profiles, and potential to regenerate GLUT1+ tumors containing the HemSC and the differentiated derivatives^6^,^10^,^14^,^16^. A rare subset (0.1–2%) of IH cells that express endothelial cell markers (such as platelet endothelial cell adhesion molecule-1 [PECAM-1/CD31], vascular endothelial-cadherin [VE-cadherin/CD144], CD34, and KDR/VEGFR-2/CD309 co-express the CD133 stem cell marker^6^. The identification of this subset suggests the existence of intermediate phenotypes (transitioning progenitor populations of interest) and stem cell heterogeneity.

We hypothesized that the HemSC is a vascular stem/progenitor cell whose proliferation is dysregulated, but not a fully transformed cell, which orchestrates IH pathophysiology through multiple signaling and regulatory networks. Therefore, to understand the process of hemangioma-genesis we characterized the progenitor hemangioma-derived stem cell (HemSC) and its lineage and non-lineage specific derivatives (Fig. 1). The results of our comprehensive phenotypic and gene expression analysis and the characterization of the tumorigenic potential of HemSCs *in vitro* show that IH derives from a dysregulated stem cell that remains in an immature, arrested stage of development.

## Results

### Molecular Signature

HemSCs have the potential to self-renew and give rise to differentiated cell types (derivatives) *in vivo* and *in vitro*^5^,^6^,^10^,^14^,^16^,^22^,^23^,^24^,^25^. To analyse HemSC gene expression and identify specific molecular signatures, CD133 expressing cells were isolated from specimens of IH immediately after surgical excision as described in Materials and Methods. Consistent with previous reports, the HemSC was identified by flow cytometry as a rare CD133+ subset representing approximately 1% of the heterogeneous population. These cells were grown *in vitro* and subjected to gene expression analysis by qPCR for genes that encode the surface markers of endothelial, fibroblast, and mast cells – key cellular components of IH. At passages 3 and 7 in culture, the expression profile of these cells was heterogeneous and no longer pure, recapitulating the heterogeneity of the original tissue (data not shown).

Using fluorescence-activated cell sorting (FACS), HemSCs were enriched and sorted for gene expression experiments as described in Materials and Methods. Prior to microarray analysis, HemSCs were identified by Prominin expression (high levels of PROM1 [prominin-1] and PROM2 [prominin-2]), the genes that encode the CD133 cell surface marker proteins, and by high expression of GLUT1 (IH clinical biomarker)^17^,^18^. Relative to normal endothelial cells (HUVEC), GLUT1^High^ HemSCs were characterized by higher expression of genes involved in vasculogenesis/angiogenesis, normal and tumor development (e.g. Angiogenesis, “Angiocrine”^26^, Stem Cell, TGFβ, Notch, HIFs, Tyrosine Kinases, Hypoxia Signaling, Tumor Metastasis, MAP Kinases, NFκB, PI3K-AKT, Homeobox, Ubiquitin-Ring/Zinc Finger domain). Specifically, relative to Hem derivatives, HemSCs expressed a significantly higher number of genes categorized as transcriptional regulators (tripartite motif [TRIM] proteins); epigenetic regulators harnessing the epigenetic machinery (zinc finger proteins, ubiquitin ligases and chromatin modifiers); long non-coding RNAs (lncRNAs); and genes of unknown function (GEO accession number GSE34989).

**Figure 2:**
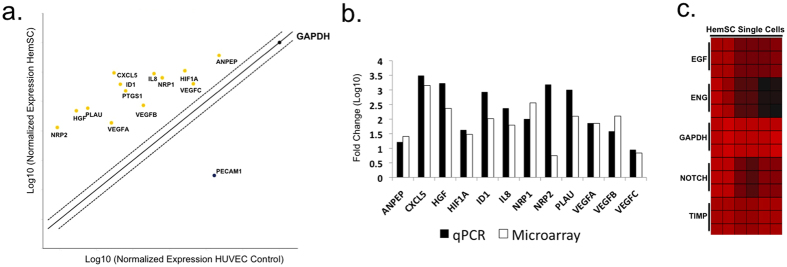
Gene expression analysis of angiogenesis signaling. Agilent whole genome oligo microarrays (one-color – Cy3 labeling) and 96-well qPCR arrays (SABiosciences) were used to examine expression profiles of genes in multiple signaling pathways involved in vasculogenesis/angiogenesis, normal and tumor development (**a**) qPCR analysis. 96-well qPCR arrays (SABiosciences) were used to examine expression profiles of genes in sorted cell populations. Fold change/regulation was calculated using delta delta C_t_ method. The values were compared to fold change gene expression analysis using one-way analysis of variance (ANOVA) of samples (Partek Genomics Suite software) (Supp. Fig. 1). The scatter plot compares the normalized expression of significant genes on the array between HemSC and HUVEC (control) to exhibit large gene expression changes. The central line indicates unchanged gene expression (selected fold regulation threshold boundary set at 2). Data points in the upper left and lower right sections meet the selected fold regulation threshold. (**b**) Microarray and qPCR analysis of gene expression in HemSC relative to HUVEC. Microarray gene expression analysis was performed using one-way analysis of variance (ANOVA) of samples to compare the normalized expression of significant genes (in the angiogenesis signaling gene panel) between HemSC ^GLUT1+^ and HUVEC (control). The gene list (Table 1) was created with specified criteria: 1) size of change defined as a fold change; and 2) significance of change defined as p-value with False Discovery Rate (FDR). qPCR analysis served as confirmation (Table 2). Fold change variations may be due to gene variants (for example, NRP2 reflects transcript variant 2 for qPCR [NRP2 variant 2, NM_003872] and transcript variant 1 [NRP2 variant 1, NM_201266] for microarray). (**c**) Single-cell gene expression. Single-cell gene expression profiles were characterized by a HT approach, fluorescence-activated cell sorting using Aria II into the wells of 96-well plates containing Platinum Taq reverse transcriptase, polymerase master mix (Invitrogen) and primers for each gene target (SABiosciences) per Fluidigm Protocol 41. The heat map represents the threshold C_t_ values (red indicates high expression). The rows correspond to the evaluated genes and columns correspond to individual HemSCs. The gene expression intensity data images were generated with Partek Genomics Suite Software.

**Figure 3:**
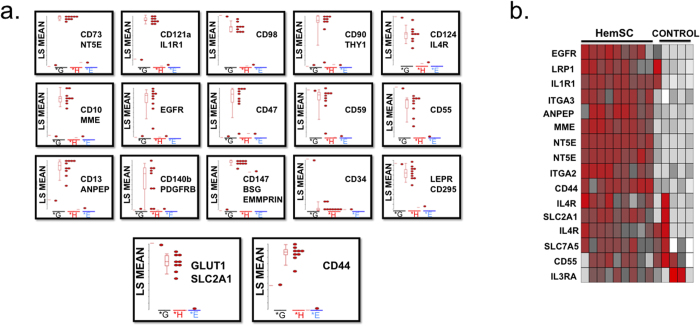
Microarray analysis of cell surface markers. Agilent whole genome oligo microarrays (one-color – Cy3 labeling) were used to examine expression profiles of 262 cell surface marker genes and SLC2A1 (GLUT1, IH clinical biomarker). The dataset for this analysis represents a subset of the original experiment and includes only HemSC ^GLUT1+^, and normal and neoplastic SC controls (HUVEC, Bone Marrow SC, Cord Blood SC, Mesenchymal SC, and Glioblastoma CSC). Microarray gene expression analysis was performed using one-way analysis of variance (ANOVA) of samples (Partek Genomics Suite software) to compare the normalized expression of significant genes (in the cell surface marker gene panel) between HemSC ^GLUT1+^ and HUVEC (control). The gene list (Table 3) was created with specified criteria: 1) size of change defined as a fold change; and 2) significance of change defined as p-value with False Discovery Rate (FDR). (**a**) Microarray gene significance dot plot. The dot plots compare the expression of significant genes (criteria 1, size of change defined as fold change) between HemSC ^GLUT1+^ and HUVEC (control) to exhibit large gene expression changes (HemSC ^GLUT1+^ - H, HUVEC endothelial progenitor (control) - E, Glioblastoma CSC – G). (**b**) Microarray gene significance heat map. The heat map compares the expression of significant genes between HemSC ^GLUT1+^ and HUVEC (control) (criteria 2, significance of change defined as p-value with False Discovery Rate [FDR]) and exhibits the expression profiles of all controls (Bone Marrow SC, Cord Blood SC, Mesenchymal SC, and Glioblastoma CSC). Flow cytometry analysis of cell surface markers served as confirmation (Fig. 5).

**Figure 4:**
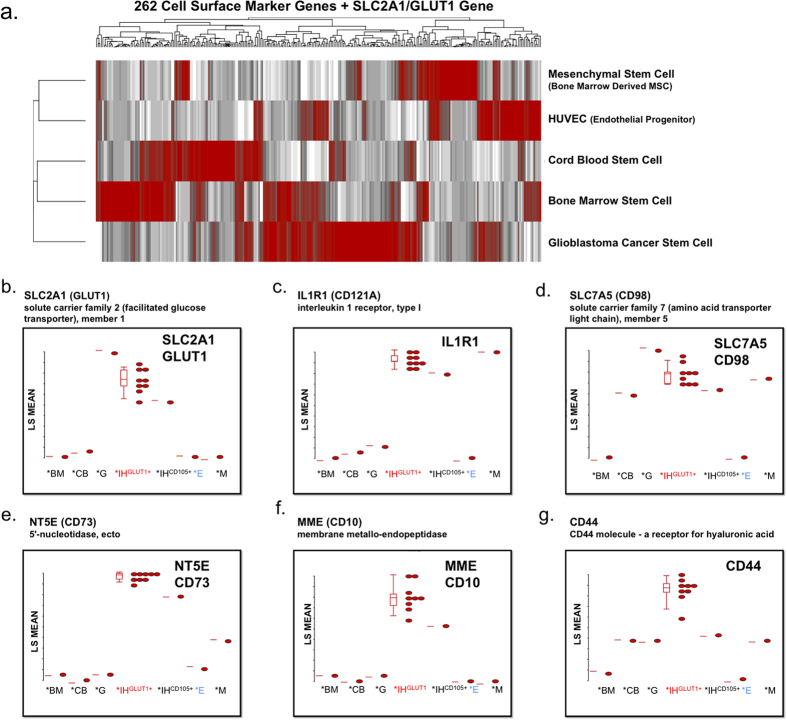
Global transcriptome of normal human stem and progenitor cell. (**a**) Hierarchical clustering analysis of human cell surface marker screening panel. Microarrays were used to examine expression profiles of 262 cell surface marker genes and SLC2A1 (GLUT1, IH clinical biomarker) normal and neoplastic SC controls (HUVEC, Bone Marrow SC, Cord Blood SC, Mesenchymal SC, and Glioblastoma CSC). The gene expression intensity data images were generated with Partek Genomics Suite Software. (**b–g**) Microarray gene significance dot plot. The dot plots compare the expression of significant genes between HemSC ^GLUT1+^ and HUVEC (control) to exhibit large gene expression changes (Bone Marrow SC - *BM, Cord Blood SC - *CB, Glioblastoma CSC - *G, HemSC GLUT1^high^ - *IH^GLUT1+^, Hem ^ENG+−^ IH^CD105+^, HUVEC endothelial progenitor (control) - *E, Mesenchymal SC - *M) of sample gene set including SLC2A1 (GLUT1 clinical marker), NT5E (CD73), ILR1, SLC7A5 (CD98), MME (CD10), and CD44.

The microarray data were validated by qPCR and single-cell gene expression analysis^27^,^28^. qPCR analysis included genes involved in vasculogenesis, proangiogenic cytokines and growth factors (Table 1 and 2; Fig. 2; Supp. Fig. 1). Notably, genes up-regulated in proliferative hemangiomas relative to the normal endothelial cell control (HUVEC) include vascular growth factors and receptors (ANPEP, FIGF [VEGFD], HGF, KDR, NRP1, NRP2, PGF, VEGFA, VEGFB, VEGFC), cytokines (CXCL5, CXCL8/IL8), adhesion molecules including NRP1, NRP2, extracellular matrix proteins and proteases (PLAU), and transcription factors (HIF1α). We also analyzed the microarray data for cell surface biomarkers (Table 3; Figs 3 and 4). In addition, our microarray gene expression analysis and qPCR validation studies showed high levels of “pro-adipogenic” and anti-inflammatory response genes (such as proliferator-activated receptor gamma [PPARγ]) in the IH samples compared with HUVEC control^29^,^30^. At the single-cell level, HemSCs showed variability in gene expression. For example, they showed homogeneous gene expression of the NOTCH, TIMP, EGF, and GAPDH genes; in contrast, Endoglin (ENG/CD105) gene expression was highly heterogeneous, ranging from high expression levels to no expression. These data were confirmed at the single-cell level by comparison of phenotypic expression (flow cytometry and bioimaging) of HemSC-specific cell surface markers (Fig. 2c).

Our microarray data include normal human stem and progenitor cell subsets that can serve as a reference as human transcriptional controls for biomarker analysis, drug-targeting pathways, and for future studies. They provide a reference for global gene expression in different cell types, developmental stages, diseases, and experimental conditions such as regenerative medicine and reprogrammed human induced pluripotent [hiPS] subsets (Fig. 4). Of the sample biomarker gene set analyzed, SLC2A1 (GLUT1, clinical marker of IH), NT5E (CD73), MME (CD10), and CD44 were over-expressed compared to the normal progenitor cell controls (HUVEC, bone marrow SC, cord blood SC, and mesenchymal SC). In contrast, ILR1 and SLC7A5 (CD98) were also expressed in normal progenitor cell controls (mesenchymal SC and mesenchymal SC/cord blood SC, respectively). SLC2A1 was over-expressed in the glioblastoma cancer stem cell (CSC) control as well. The gene expression of mTOR (mammalian target of rapamycin), a drug-target, showed high variability (Supp. Fig. 2a). However, Protein Kinase B (AKT1), a kinase upstream of mTOR, was consistently over-expressed. The PPAR/Adipocytokine signaling pathway microarray analysis showed significant over-expression of transcription factors (PPARD, PPARG, KLF10, RXRB) and the clinical biomarker of IH, GLUT1 (SLC2A1) (Supp. Fig. 2b).

### TGF-β Signaling – Endoglin (CD105) Gene Signature

Because the single-cell analysis of HemSCs showed high variability in the phenotypic (CD105) and genotypic (ENG) expression of endoglin we performed a further molecular analysis of the IH endoglin subset (Hem ^ENG+^). The global gene expression profile of Hem ^ENG+^ (100–200 Hem ^ENG+^ sorted cells) was analyzed by microarray and qPCR (data shown as CD105^+^ subset control in Fig. 4b–g). The Hem ^ENG+^ progenitor sub-population was compared with glioblastoma CSC, mesenchymal SC, bone marrow SC, cord blood SC, and HUVEC cells, which were FACS-enriched and sorted for purity before gene expression analysis. Prior to performing the gene expression studies, Hem ^ENG+^ were FACS-enriched and sorted for CD105+ subset. The results showed that the endoglin gene was expressed at high levels in the Hem ^ENG+^ sub-population. Molecular characterization of the bulk subset of Hem ^ENG+^ showed a higher expression of VEZF1 and a lower expression of PROM1/SALL4, suggesting endothelial differentiation with the involvement of TGF-β signaling. The PTGS1 (prostaglandin-endoperoxide synthase 1/COX1) gene was also over-expressed in the Hem ^ENG+^ sub-population. PTGS1 gene expression is regulated by Kruppel-like factors, zinc finger transcription factors (such as KLF10) that also act as key transcriptional regulators of TGF-β signalling^31^,^32^. Specifically, KLF10 targets the KDR promoter^31^,^32^. PTGS1 gene expression is also induced by estrogen. Angiopoietin-like 4 (ANGPTL4/FIAF), a direct glucocorticoid receptor target, was exclusively expressed at high levels in the Hem ^ENG+^ sub-population.

### Cell Surface Marker Analysis

The analysis of surface markers can provide useful information for the development of diagnostic tools and indicate novel targets for antibody-based therapy of IH. Therefore, we performed a HT screening of primary IH-derived cells using a stem cell-specific panel of 240 monoclonal antibodies to surface antigens. The results were validated by flow cytometry, microarray (Table 3; Figs 3 and 4) and qPCR analysis of three primary IH cell cultures, using HUVEC, glioblastoma CSC, bone marrow SC, cord blood SC, and mesenchymal SC as controls.

**Figure 5:**
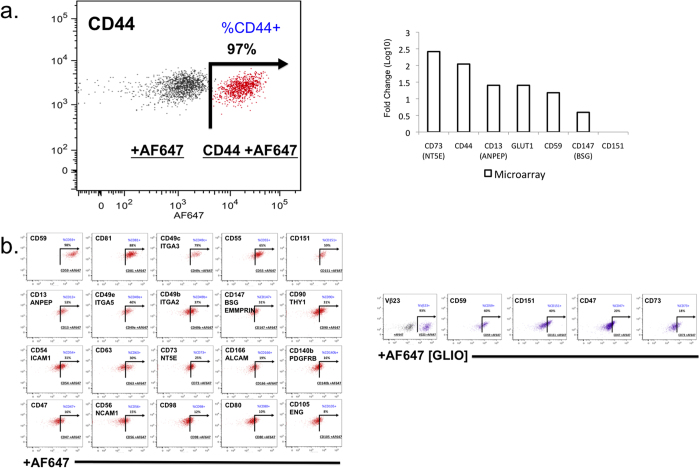
Analysis of human cell surface markers. Human cell surface markers (242 monoclonal antibody screening panel including mouse and rat isotype controls from BD Lyoplate A, Lyoplate B, Lyoplate C) were analyzed by HT flow cytometry using a BD LSRII HTS and Cellomics ArrayScan HC imaging microscope. (**a**) Flow cytometry and Microarray analysis. CD44 is highly expressed in IH cells, as shown by flow cytometry (dot plot graph overlays) and microarray analyses. Microarray gene expression analysis was performed to compare expression of a sample set of cell surface marker genes between HemSC ^GLUT1+^ and HUVEC (control). The fold change examines differences between proliferating vascular progenitor subsets (HUVEC) and proliferating dysregulated vascular progenitor tumor subsets (IH). (**b**) Flow cytometry graphs. The data represents a sample set of cell surface markers for assay development of a comprehensive panel of potential biomarkers and therapeutic targets.

The most highly expressed markers (defined as greater than 10 - AF647+ %Parent) included Alkaline Phosphatase (AP), CD10, CD13, CD44, CD46, CD47, CD59, CD73, CD105, and CD147 (Tables 4 and 5; Fig. 5). The biologic classification of IH proposed by Mulliken and Glowacki identified “the endothelium in hemangiomas as characteristic of differentiation: Weibel-Palade bodies, alkaline phosphatase, and factor VIII production”^2^. Alkaline phosphatase (AP) is regarded as a benchmark pluripotent stem cell marker^33^. CD44 is a key marker of CSC^34^, along with CD133. CD44 controls normal development by influencing cell growth, survival, and differentiation^35^,^36^. In IH, CD44 was highly expressed in both the heterogeneous and HemSC populations. CD105 (Endoglin/ENG), a co-receptor for transforming growth factor-beta (TGF-β), is involved in angiogenesis, particularly tumor angiogenesis^37^. CD13 (metalloprotease; aminopeptidase N; ANPEP/APN) is also an important regulator of angiogenesis and is overexpressed in tumor cells^36^. Within the tumor microenvironment, ANPEP expression is induced by vascular endothelial growth factor (VEGF), and high levels of CD13 have been associated with tumor progression in breast, ovarian, and prostate cancers^38^. CD10 (CALLA; Neprilysin; membrane metalloendopeptidase) is expressed in the tumor microenvironment, where it promotes endothelial cell growth and angiogenesis by mobilizing fibroblast growth factor-2 (FGF-2) and activating AKT1 signaling, a cell proliferation and prosurvival pathway^39^,^40^.

### HemSC-Derived Tumorsphere and Derivative Formation

**Figure 6:**
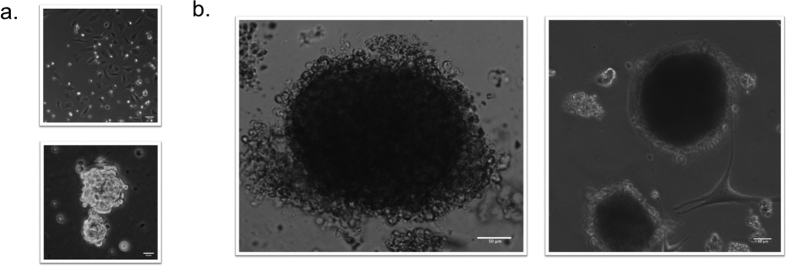
Tumorsphere and derivative formation demonstrates the HemSC tumorigenic and differentiation potential. (**a**) Tumorsphere controls. HUVEC were used as a negative control and glioblastoma CSC as a positive control. Glioblastoma CSC formed tumorspheres (lower), whereas HUVEC formed no tumorspheres (upper). (**b**) Derivative formation. HemSC-derived tumorspheres were grown as a suspension (in non-adherent ultra-low cluster plates) and then transferred onto an adherent matrix. The latter condition afforded the generation of differentiated derivatives that attached to and spread on the culture substrate. Bioimaging of HemSC-derived tumorsphere formation in non-adherent ultra-low cluster plates showed no derivative formation.

HemSCs are characterized by high expression of genes involved in vasculogenesis, angiogenesis, tumorigenesis, and associated signaling pathways. *In vitro* tumorsphere formation assays were performed to investigate the tumorigenic and differentiation potential of the HemSC-derived tumorspheres (Figs 6). The results confirmed the HemSC as a rare CD133+ subset comprising approximately 1% of the tumor cell population, with an ability to self-renew, multipotential ability to give rise to several cellular lineages with distinct morphologies and gene expression profiles, and a potential to regenerate GLUT1+ tumorspheres containing the HemSC and the differentiated derivatives (pericytes).

**Figure 7:**
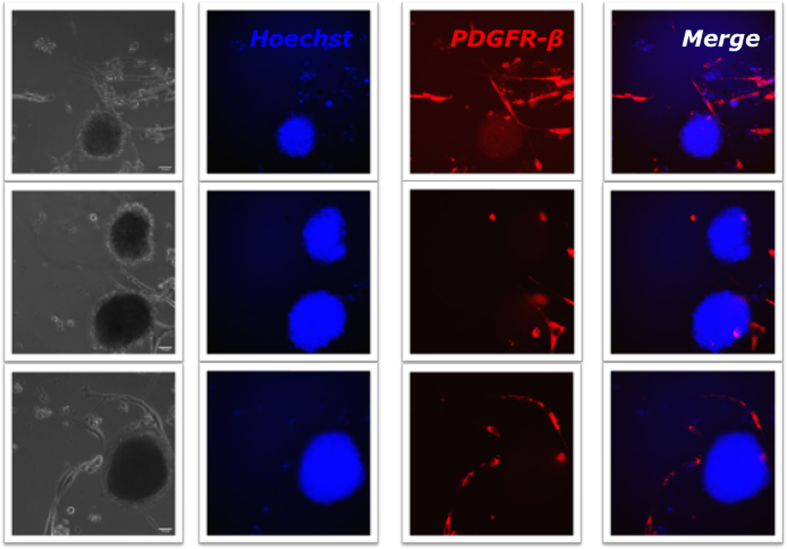
HemPericyte derivative bioimaging analysis shows *de novo* formation of derivatives from undifferentiated HemSCs. The differentiated derivatives from the tumorspheres were characterized by immunostaining for the pericyte surface marker PDGFR-β. Using the Applied Precision Personal DV live-cell imaging system, the study design included 21-h imaging studies with live imaging of HemSC-derived tumorsphere and derivative formation/interaction (Video 1,2–3). Controls comprised the use of nuclear stain, cell surface markers, specificity controls (Ms IgG2a, κ), and beads tagged with secondary antibody for exact instrument intensity measurements. HemSC-derived tumorsphere + pericytes (platelet-derived growth factor receptor β (PDGFR-β) CD140b/AF647 Positive): +Hoechst stain/+AF647/Merged. The PDGFR-β cell surface marker was used to identify the HemPericytes among the cultured derivatives. HemPericytes expressed PDGFR-β whereas the tumorspheres did not.

To regenerate tumorspheres *in vitro* and analyze their differentiation potential HemSC-derived tumorspheres were first grown as non-adherent cultures in ultra low cluster plates and then transferred onto an adherent matrix (Fig. 6). The latter condition afforded the generation of differentiated derivatives that attached to, and spread on the culture substrate. HUVEC were used as a negative control and glioblastoma CSC as a positive control. In culture, glioblastoma CSC formed tumorspheres, whereas HUVEC formed no tumorspheres under the same experimental conditions (Fig. 6a). Bioimaging of HemSC-derived tumorsphere formation in non-adherent ultra low cluster plates showed no derivative formation besides the tumorspheres. However, after transferring to an adherent matrix they showed *de novo* formation of differentiated derivatives that attached to and spread on the culture substrate (Fig. 6b). Pericytes (HemPericytes) are abundant in IH proliferating-phase tissue and represent a key cellular component^6^,^41^. Platelet-derived growth factor receptor-beta (PDGFR-β) is a pericyte surface marker^42^,^43^,^44^,^45^. Therefore, differentiated derivatives from the HemSC tumorspheres were characterized by immunostaining for PDGFR-β. A subset of adherent cells expressed PDGFR-β whereas the tumorspheres did not (Fig. 7). HT 21-h live imaging analysis of 13 distinct HemSC-derived tumorspheres showed *de novo* formation of pericyte derivatives from undifferentiated HemSCs, and a dynamic interaction between the HemSC -derived tumorsphere and its derivative population (with active movement and engagement of the derivative population) (Video 1,2–3). This finding showed that the *de novo* formation of pericyte-like cells originated from a heterogeneous population of stem cells in the tumorspheres, and that the HemSC is a multipotent stem cell arrested at an early stage of development (incompletely differentiated) with an ability to differentiate into several cellular lineages (lineage and non-lineage specific differentiation).

**Figure 8:**
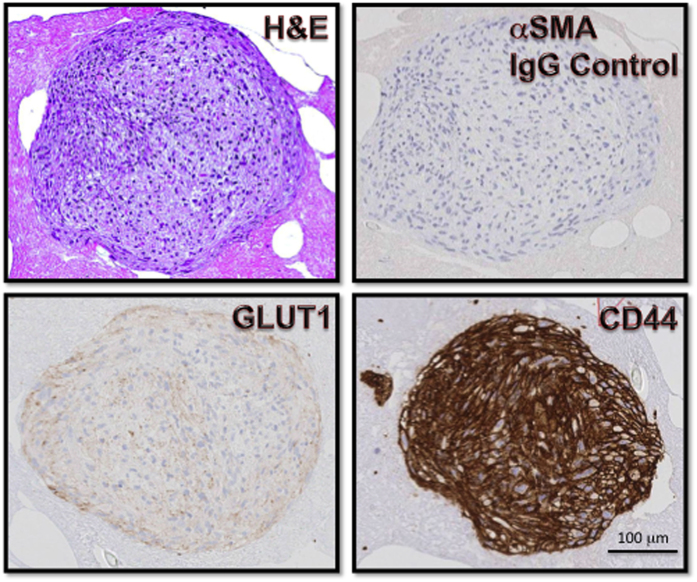
Immunohistochemical analysis of HemSC-derived tumorspheres. Tumorspheres were characterized for GLUT1 and CD44 by immunohistochemistry as described in Methods.

**Figure 9:**
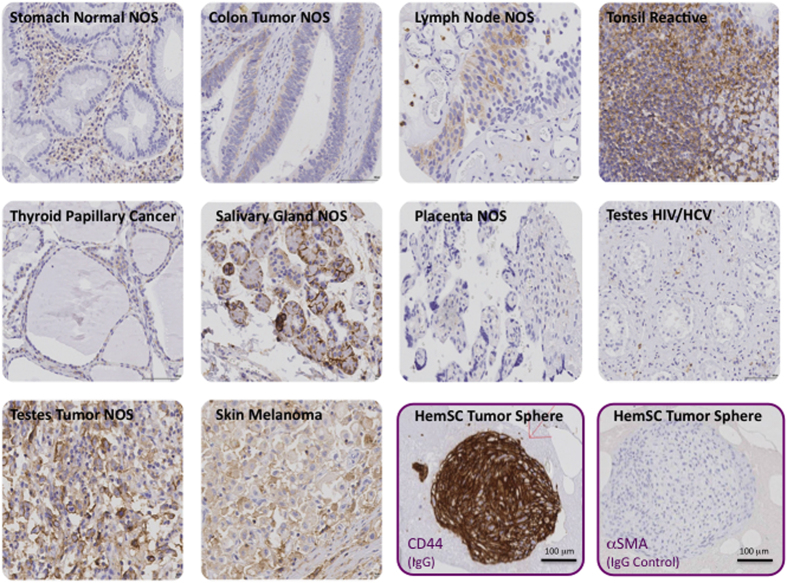
Immunohistochemical analysis of CD44 in human tissues. A human tissue array including the indicated tissues was analysed by immunohistochemistry with antibody to CD44 as described in Methods. Alpha smooth muscle actin antibody was used as a negative control.

The HemSC-derived tumorspheres were characterized by flow cytometry and immunohistochemistry (Fig. 8). Flow cytometry analysis of pooled tumorspheres showed a heterogeneous population, similar to the population derived from monolayer culture of primary IH cells. HemSC was identified as a rare CD133+ subset comprising approximately 1% of the heterogeneous population, a frequency similar to that of HemSCs directly isolated from IH specimens. The immunohistochemical characterization of the HemSC-derived tumorspheres showed expression of GLUT1, the diagnostic marker of IH, as well as high levels of CD44, a well-established tumor stem cell marker. CD44 is highly expressed in IH cells, as shown by our genotypic and phenotypic analyses, and can be detected by immunohistochemistry in various normal and neoplastic tissues such as lymph nodes, melanoma, tumors of the testis. However, we did not find CD44 immunoreactivity in placenta (Fig. 9). Thus, the HemSC-derived tumorspheres recapitulated *in vitro* the gene expression pattern of IH.

## Discussion

The data presented show that IH originates from a dysregulated but not fully transformed multipotent stem cell. IH is characterized by high expression of genes involved in vasculogenesis, angiogenesis, tumorigenesis, and associated signaling pathways. The identification of transcriptional regulator genes and analysis of gene expression at the single-cell level suggests the existence of intermediate phenotypes, unique subsets, and stem cell heterogeneity. Moreover, our observations point to an important role of the immune system in hemangioma-genesis, the strategies used in the proliferative phase of IH formation to evade immunosurveillance, and the potential benefits of immunotherapy. Our data also show that HemSC have the potential to regenerate GLUT1+ tumorspheres from which differentiated derivatives originate. The *de novo* formation of pericyte-like derivatives reflects the heterogeneity of a hemangioma-derived multipotential stem cell arrested at an early stage of development (incompletely differentiated) with the ability to differentiate into several cellular lineages (lineage- and non-lineage specific differentiation). Finally, we provide a comprehensive panel of potential biomarkers and drug-targets (which can be further explored for assay development), and propose novel potential therapeutic strategies for IH, identified by our comprehensive analysis.

Signature genes that differentiate HemSCs from the Hem-derivatives include over-expression of transcriptional regulators that encode zinc finger transcription factor proteins such as SALL4, tripartite motif (TRIM) proteins (RING type E3 ubiquitin ligases), and long non-coding RNAs (lncRNAs). Zinc finger genes, found in 3% of the human genome, encode proteins that serve as transcriptional regulators^46^,^47^. TRIM proteins regulate nuclear receptors, and have been associated with tumorigenic pathways and hormone (estrogen) responsive cancer cells^48^. Long non-coding RNAs (RNA transcripts with a regulatory function) are localized either in the nucleus or in the cytoplasm, where they are involved in transcriptional or post-transcriptional regulation^30^,^49^,^50^. For example, in HemSCs, the zinc finger transcription factor SALL4 is over-expressed. SALL4 is a stem cell factor normally restricted to human embryonic stem cells (hESCs) and somatic stem cells^16^. The zinc finger transcription factor vascular endothelial zinc finger 1 (VEZF1) is over-expressed in Hem-derivatives, HUVEC, and cord blood SCs. VEZF1 is normally expressed in endothelial cells during vascular development^51^,^52^. A rare subset of HemSCs that express the endothelial marker PECAM-1 co-express the transcription factors SALL4 and VEZF1. The identification of this subset further suggests the existence of intermediate phenotypes.

Gene expression is a dynamic process^53^,^54^. The variability observed at the single-cell level reflects true differences, as opposed to averaged measurements of a large number of cells such as those obtained by microarray and qPCR. Our microarray and qPCR analysis of Endoglin showed homogeneous expression that did not reflect the actual heterogeneity occurring at the single-cell level. Our qPCR/microarray analysis of bulk gene expression in 100–200 flow sorted HemSC showed that HemSCs differ from control HUVEC and are characterized by low expression of endoglin. However, the analysis of HemSC gene expression at the single-cell level (also validated by flow cytometry and bioimaging) clearly identified a heterogeneous expression pattern of the endoglin (ENG) gene, revealing a unique subset of cells (HemSC ^ENG+^) and stem cell heterogeneity, with ENG expression levels varying from high to low/non-detectable. The identification of HemSC ^ENG+^ and HemSC ^ENG-^ progenitor subsets suggests the existence of intermediate phenotypes (transitioning progenitor populations of interest); a hemangioma stem cell (hemangioma hemangioblast) dysregulated/arrested in development - differing from multiple stem cells (such as neural crest SC, bone marrow SC, cord blood SC, mesenchymal SC) heterogeneously dysregulated/arrested at varying stages of development; and/or a stem cell marker that reflects tumor heterogeneity (the inactive normal stem cell *vs*. the active neoplastic stem cell involved in vascular pathogenesis [hemangioma-genesis]).

The endoglin gene encodes a transmembrane auxiliary receptor for TGF-β^55^. TGF-β signaling is important in normal vascular development (vasculogenesis, angiogenesis, and embryonic vascular assembly) and plays a role in tumor angiogenesis^32^,^55^,^56^,^57^,^58^,^59^,^60^,^61^. In early development, endoglin is required for hemangioblast specification, is a marker for an embryonic progenitor, the hemangioblast (in addition to the hemangioblast marker KDR/VEGFR-2/CD309), and serves as an important regulator of hematopoietic and endothelial lineage commitment^56^,^57^,^58^,^59^,^62^,^63^,^64^,^65^,^66^,^67^. Endoglin expression levels are heterogeneous in different cell sub-populations. For instance, during yolk sac development, blood cells are characterized by low expression, whereas endothelial cells present high expression of this receptor^56^. In mouse embryonic stem cells-derived embryoid bodies endoglin marks the hemangioblast on day 3 of differentiation, and in the absence of endoglin (Eng^−/−^) there is a significant reduction in hemangioblast frequency. Eng-null (Eng^−/−^) mice die by embryonic day 10 due to abnormal vasculature development^56^,^68^. Within normal tissue, endoglin is highly expressed in active vascular endothelial cells during embryogenesis and in syncytiotrophoblasts of term placenta, and is expressed at low levels in resting endothelial cells^55^. In active, normal endothelial cells, endoglin expression is required for TGF-β receptor ALK1 (activin A receptor type II-like 1/ACVRL1) signaling, which promotes proliferation, migration, and angiogenesis^69^. ALK1 expression indirectly inhibits ALK5 (TGF-β receptor 1/TGFβR1/ TGFR1). In *resting* (quiescent) endothelium, endoglin is expressed at low/non-detectable levels. Thus, TGF-β/ALK5 signaling inhibits cell proliferation and migration. In vascular pathology, endoglin expression is highly expressed and upregulated in tumor endothelium, placenta-derived cells (in pre-eclampsia), and endothelial cells in response to vascular injury^32^,^55^. Endoglin expression is regulated and stimulated by hypoxia and TGF-β signaling pathways^70^. Further single-cell characterization of the HemSC ^ENG+^ (PROM1^High^/ENG^High^) and HemSC ^ENG−^ (PROM1^High^/ENG^Low^) sub-populations, and analysis of the endoglin zinc finger/promoter domain (SP/KLF)^32^ can differentiate the hemangioma hemangioblast from the derivative population, the HemSC (dysregulated subset) from the other stem cells present in the vascular compartment, and the inactive normal stem cell *vs.* the active neoplastic stem cell.

Several observations point to an important role of the immune system in hemangioma-genesis. The initial stage of IH proliferation generally coincides with the onset of maturation of the immune system (at age 6–12 months), and a significant number of CD8+ T cells have been identified in IH^71^,^72^. During the transition from the proliferation to the involution phase of IH, the immune system can also play a role with variation in indoleamine 2,3-dioxygenase (IDO) expression levels^72^. Understanding the specific crosstalk between the host immune system and IH during these stages of tumor development is important; for example, to identify the strategies HemSCs use to evade immunosurveillance and the potential benefits of immunotherapy. We analyzed the hemangioma cell surface proteome by a comprehensive stem cell surface marker screening panel including monoclonal antibodies to molecules that modulate the immune response. Based on our results and data published in the literature, an interplay can be envisaged between the immune system and hemangioma-genesis. IH arises at the time of transition from immunotolerance to immunocompetence, which can enhance the growth of IH. The onset of immunocompetence at birth coincides with a transition to a higher number of adult immune stem cells than fetal immune stem cells^73^,^74^,^75^,^76^,^77^. Our analysis of the cell surface markers in the proliferative phase of IH formation reflects mechanisms to escape immunosurveillance. CD47, “a ‘don’t eat me’ signal for phagocytic cells” is expressed on the surface of IH cells as well as on “all human solid tumor cells”^78^. In addition to the ‘don’t eat me’ sirens, tumors use several alternative strategies to evade immunosurveillance and a variety of these can be identified in IH. The generation of adenosine by CD73, highly expressed by IH, suppresses the T cell response within the tumor microenvironment^79^. This immunosuppressive effect promotes tumorigenesis^79^,^80^,^81^. CD59 and CD46, also highly expressed in IH, prevent complement-mediated tumor cell lysis^82^. CD59 (Protectin) serves as a line of defense, acts against complement-mediated lysis by incorporating into a membrane attack complex (MAC), and is regarded as a powerful inhibitor of complement cytolysis. CD46, a complement regulatory protein, inhibits complement activation and serves to protect the host cell against attack^82^. Additional cell surface markers included CD10 (zinc-binding metalloendopeptidase and diagnostic pediatric lymphoma and leukemia cell surface marker)^36^,^39^,^40^, and CD13 (zinc-binding metalloprotease aminopeptidase, pericyte cell surface marker, and myeloid [normal and neoplastic] cell surface marker)^36^,^38^.

We studied tumorsphere formation by HemSCs to investigate their tumorigenic and differentiation potential. CD44 served as a highly expressed cell surface marker to validate the expression profile in the HemSC-derived tumorsphere and tissue array analysis. We used PDGFR-β, a highly specific pericyte marker^83^, to identify the HemPericytes among the cultured derivatives. Our analysis revealed abundant PDGFR-β+ HemPericytes. In normal vascular development, endothelial cells and pericytes are derived independently with distinct lineages^20^,^21^ although there are “intimate interactions between endothelial cells and pericytes”^44^. During angiogenesis, PDGFR-β+ cells are attracted to the vascular endothelium and secrete vascular endothelial growth factor (VEGF)^43^,^44^. Pericytes control endothelial cell proliferation and differentiation via a paracrine and cell-cell contact mechanism, and take an active role in angiogenesis as a survival factor. Thus, HemSC-derived pericytes may serve as a survival factor for the proliferating IH. Importantly, the *de novo* formation of pericyte-like derivatives reflects the stem cell heterogeneity of HemSCs in the tumorsphere (including undifferentiated perivascular mesenchymal cells among other stem cells) and a hemangioma-derived multipotential stem cell arrested at an early stage of development (incompletely differentiated) with an ability to differentiate into several cellular lineages (lineage- and non-lineage specific differentiation).

The results of our study provide insight into the cellular and molecular mechanisms underlying the complex clinical diversity in IH; however, the limited number of our patient samples is insufficient to assess the clinical variability for classification. We used cellular, molecular, and genetic techniques - in particular flow cytometry - for assay development of a comprehensive panel of potential biomarkers and drug-targets. Our specific panel (Table 6) can be used for future studies of large cohorts for biomarkers and therapeutic targets (existing or novel drugs); the construction and analysis of gene regulatory networks; the characterization of hemangioma-derived stem cell subsets by established methods of multiparameter analysis (e.g. the identification of multiple cell surface markers will afford polychromatic flow cytometry [PFC]^84^ investigation to further characterize dysregulated HemSCs); the study of IH-derived stem cell subsets by novel techniques and applications (e.g. isoform sequencing platforms for novel genes/gene isoforms and single-cell transcripts); the dissection of IH heterogeneity (including different cell types) for clinical/pathological classification; and the development of novel precision medicine isogenic models, such as patient-derived human induced pluripotent stem cells for a precision medicine approach to the treatment of the individual patient. For practical applications, an antibody panel can be designed to identify expression of GLUT1 (diagnostic clinical marker for IH) and IH cell surface signature markers (such as CD10, CD13, CD44 and CD73) by immunohistochemistry, an established method routinely used for *in vitro* diagnostic assays and a useful adjunct to diagnostic histology.

Besides surgical excision, current pharmacological treatments for IH, corticosteroids or propranolol, are administered for several months and have adverse effects. Rapamycin, an mTOR inhibitor, has been shown to inhibit HemSC self-renewal and vascular differentiation potential in patient-derived hemangioma stem cells^85^. Our microarray data for HemSCs GLUT1^High^ showed high variability in mTOR gene expression but significant over-expression of AKT1 (one of the upstream kinases in adipocytokine signaling pathways). Our gene expression analysis show high levels of PPARγ, a “pro-adipogenic” and anti-inflammatory response gene. The expression of the placental glucose transporter (such as GLUT1) is regulated by glucocorticoids (adrenal steroids) and the nuclear receptor PPARγ (which interacts with estrogen to regulate and promote adipogenesis)^30^,^86^. Researchers have performed quantitative studies to investigate the role of “pro-adipogenic” genes (such as PPARγ) in hemangioma-genesis, proliferation to involution, and their interaction with propranolol, a beta-blocker used in the treatment of IH^29^. Targeting the adipocytokine/PPAR signaling pathway affords an alternative therapeutic strategy to challenge the fibrofatty differentiation of IH, which can result in life-long, function-compromising effects including disfigurement. CD73 serves as a potential biomarker and drug target for IH, with the adenosine pathway serving a critical role in immune activation and inflammation^87^. To target the immunosuppressive effects that promote tumorigenesis^79^,^80^,^81^, immune response modifiers, such as humanized monoclonal antibody to CD73, can serve as a complementary treatment. For instance, a humanized monoclonal antibody to CD73 may inhibit IH growth by blocking CD73 and stimulating the activity of T cells.

In conclusion, our data show that IH originates from a dysregulated but not fully transformed multipotent stem cell, which orchestrates IH pathophysiology through multiple signaling and regulatory networks. The potential biomarkers we identified can afford the development of diagnostic tools and precision-medicine therapies to “rewire”^88^ or redirect cellular transitions at an early stage, such as signaling pathways or immune response modifiers. Our investigative approach can be used to characterize dysregulated progenitor cell subsets involved in other diseases and identify druggable targets for their treatment. For example, our microarray data include normal human stem and progenitor cell subsets. This information provides a valuable tool for researchers to evaluate biomarker analysis and drug-targeting pathways, as well as a reference for studies of global gene expression in different cell types, developmental stages, diseases (e.g. investigating progenitor cells in an inflammatory environment), and cell-based therapies such as for regenerative medicine.

## Methods

### Cells and Culture Media

The primary cells used were kind gifts from Dr. June Wu’s Laboratory (Columbia University; IH HemSCs H41, H48, H50, H52, H53) and Dr. Viviane Tabar’s Laboratory (Memorial Sloan-Kettering Cancer Center; Glioblastoma Cancer Stem Cells [CSC]). The control cells (all purchased from Lonza) include the following: 1) early passage, normal human umbilical vein endothelial cells (HUVEC); and 2) early passage, normal progenitor/stem cells (bone marrow CD133+ [bone marrow SC]; cord blood CD133+ [cord blood SC]; and mesenchymal CD133+ [mesenchymal SC]). The HemSC were isolated as follows. Specimens of IH were obtained during surgery and a pathologist confirmed the diagnoses. HemSCs were isolated from these specimens immediately after surgical excision as described by Khan *et al*.^14^. IH samples were minced with a scalpel and digested using collagenase (Roche) to obtain a single-cell suspension. Cells expressing CD133 were isolated using the magnetic microbead cell sorting system (Miltenyi) and seeded onto adherent fibronectin coated plates (BD Biosciences) (in EBM-2 medium supplemented with 20% fetal bovine serum [FBS] and penicillin/streptomycin [Lonza]). When 60–80% confluency was reached, the cells were passaged and seeded on adherent fibronectin/matrigel coated cell culture plates (BD Biosciences) and non-adherent ultra-low attachment cell culture plates (Corning) for HemSC-derived tumorsphere formation. Prior to gene expression experiments, early passage, pure HemSC – CD133 (Miltenyi) positive cells were isolated by fluorescence-activated cell sorting.

### HUVEC, CSC and Progenitor/Stem Cell Controls

Similar to HemSC cell culture conditions, the HUVEC, CSC, and progenitor/stem cell controls were seeded onto fibronectin coated plates (BD Biosciences) (in EBM-2 medium supplemented with 20% FBS and penicillin/streptomycin [Lonza]).

### Flow Cytometry/Bioimaging Analysis

All antibodies were purchased from BD Biosciences or Miltenyi Biotec. Monoclonal antibodies were available in lyoplate format (BD Biosciences lyoplate human cell surface marker screening panel) or in stabilizer buffer (pure CD133-1/2, Miltenyi Biotec), and directly conjugated with AlexaFluor 647, with the following exceptions of fluorochrome conjugated antibodies: CD133-1/2 (AC133/293C3); CD31 (AC128); CD105 (43A4E1) conjugated with fluorescein isothiocyanate (FITC), R-phycoerythrin (PE), or allophycocyanin (APC) (depending on study design). Antibody staining and flow cytometry/bioimaging analysis were performed following the manufacturers’ protocols. Cells were grown in suspension or as an adherent monolayer. For uniform labeling, cells were 1) harvested, 2) resuspended in washing and staining buffer (Miltenyi MACS BSA buffer and autoMACS rinsing solution or BD Biosciences BD Pharmingen stain buffer [FBS] with FcR Blocking Reagent [Miltenyi]), and 3) labeled as a single-cell suspension. Cells in adherent culture plates were detached (with Trypsin-EDTA [Corning] or BD Cell Recovery Solution/Accutase Cell Detachment Solution [BD Biosciences]). Compensation was done in multi-color stainings using single color staining of the cells and compensation beads (BD Biosciences). The cells were analyzed by HT flow cytometry using a BD LSRII HTS and Cellomics ArrayScan HC imaging microscope.

### Flow Cytometry Gating Strategy and Sorting Technique

Dead cells were excluded using Forward Scatter (FSC)/Side Scatter (SSC) parameters and viability dyes (4′,6-diamidino-2-phenylindole [DAPI FluoroPure (Invitrogen)], Propidium Iodide [PI (Invitrogen)], or Hoechst [Hoechst 33342 (BD Pharmingen)]). Viable cells were further gated as a singlet population using doublet discrimination parameters. The graphs (histograms and dot plots) display data from the analysis of the region of interest (ROI) based on the defined gating strategy parameters. Histogram and dot plot overlays include specificity controls and were prepared with FlowJo software (FlowJo, LLC).

Using the BD Aria II instrument, cells were sorted directly into: 1) BD Falcon 96-well microplates for high-throughput flow cytometry analysis (HTS plates), 2) BD Falcon 96-well black/clear microplates for bioimaging, 3) 200 μL SuperAmp tubes with 6.4 μL SuperAmp Lysis Buffer (Miltenyi Biotec) for microarray and qPCR analysis, and 4) MicroAmp Optical 96-Well Reaction Plates (Invitrogen) with Platinum Taq reverse transcriptase (Invitrogen), polymerase master mix (Invitrogen) and primers for each gene target (SABiosciences) in each well for single-cell gene expression analysis.

### Tumorsphere Bioimaging

Using the Applied Precision Personal DV live-cell imaging system, the study design included 21-h imaging studies with live imaging of HemSC-Derived tumorsphere and derivative formation/interaction. Controlled studies included use of nuclear stain, cell surface markers, the specificity controls (Ms IgG2a, κ), and beads tagged with the secondary for exact instrument intensity measurements.

## Phenotypic Analysis

### HTS Cell Surface Marker Analysis

Cell Surface Marker Screening of Heterogeneous Cell Populations (H41, H50, H52, H53) - LyoplateA/B/C- Early passage IH cells were analyzed and flow sorted using the BD Aria II (BD Biosciences) instrument, FACSDiva (BD Biosciences) and FlowJo software (FlowJo, LLC); appropriate compensation studies were conducted. Using a HT approach, 10,000 cells were sorted directly into 96-well plates, introduced to 20 μL/well of purified antibodies, conjugated with Alexa Fluor 647 detection reagent, and fixed with CytoFix fixation buffer (BD Biosciences). Prior to analysis, samples were stained with DAPI FluoroPure (Invitrogen) nucleic acid stain. The profiles of the human cell surface markers (242 antibody screening panel) were analyzed by HT flow cytometry using the BD – HT sampler instrument (LSRII HTS) and high-content bioimager, Cellomics ArrayScan HC (high-content and high-throughput) imaging microscope.

Cell Surface Marker Screening of HemSCs - Early passage cells were analyzed and flow sorted using the BD Aria II (BD Biosciences) instrument, FACSDiva (BD Biosciences) and FlowJo software (FlowJo, LLC). Appropriate compensation studies were conducted. Using a HT approach, 500 CD133+/CD31−cells (Miltenyi) were sorted directly into 96-well plates and introduced to 20 μL/well of purified antibodies, conjugated with AlexaFluor 647 detection reagent (BD Biosciences), and fixed with CytoFix fixation buffer (BD Biosciences). The profiles of the human cell surface markers (high expression markers from heterogeneous cell population) were analyzed by HT flow cytometry using the BD LSRII HTS instrument.

## Gene Expression Analysis

### HT Gene Expression Analysis

Prior to gene expression analysis, early passage IH (H41, H48, H50, H52, H53) HemSCs (progenitor) and differentiated cells (derivatives), glioblastoma CSC, mesenchymal SC, bone marrow SC, cord blood SC, and HUVEC cells were FACS enriched and sorted for purity. The microarray data were confirmed by qPCR and single-cell gene expression for the highest expressing genes for precise measurement. In addition, HemSC gene expression data were confirmed by comparison of phenotypic expression of HemSC specific cell surface markers. The microarray data are available at the GEO database (http://www.ncbi.nlm.nih.gov) - GEO accession number GSE34989.

### Microarray

The cells were flow sorted directly into Miltenyi SuperAmp lysis buffer for SuperAmp preparation kit, and RNA amplification was performed on a global PCR protocol. The mRNA was isolated using magnetic bead technology; 250 ng of amplified cDNA was used for gene expression analysis (~40,000 genes) using Agilent whole genome oligo microarrays (one-color – Cy3 labeling). Data analysis was performed with Gene Set Enrichment Analysis (GSEA) software (with standard gene set as permutation type, 1,000 permutations and log2 ratio of classes as metric for ranking genes) and Partek Genomics Suite software. With the Partek Genomics Suite software, data were normalized and gene expression analysis was performed using one-way Analysis of Variance (ANOVA) of samples. The gene list was created with specified criteria: 1) size of change defined as a fold change > 1.5 or fold change <-1.5; and 2) significance of change defined as p-value with False Discovery Rate (FDR) <0.05 or p-value with False Discovery Rate (FDR) <0.1. The gene expression intensity data images were generated with Partek Genomics Suite Software.

### qPCR

Quantitative real-time PCR was performed with an Applied Biosystems 7300 instrument using SYBR Green PCR Master Mix (Qiagen). 96-well PCR arrays (SABiosciences) were used to examine expression profiles of genes in sorted cell populations. Per manufacturer’s instructions: per well (25.0 μl final volume) = 12.5 μl RT^2^ SYBR Green qPCR Mastermix (#330520)/10.5 μl ddH2O/1.0 μl cDNA (up to 250 ng)/1.0 μl (10 μM) PCR primer pair assay; thermal profile protocol is specific for Applied Biosystems 7300 instrument: Stage 1: 95 °C, 10 min; Stage 2: 40 cycles of (95 °C, 15 sec; and 60 °C, 60 sec). For data analysis, fold regulation cut-off was set as 2 and the methods used for housekeeping genes (HKG)/internal controls included a list of genes selected from the entire 96 well plate including 5 HKG that had small changes in the threshold cycle (CT) values across all samples. C_t_ values for these genes were then geometrically averaged and used for the ΔΔCT calculations.

### Single-Cell Gene Expression

To survey gene expression profiles by a HT approach, fluorescence-activated cell sorting was performed using BD Aria II, 100 cells per well and for single-cell samples 1 cell directly flow sorted into the wells of 96-well plates containing Platinum Taq reverse transcriptase, polymerase master mix (Invitrogen) and primers for each gene target (SABiosciences) as per Fluidigm manufacturers Protocol 41 instructions and as described in the Nature Methods workflow for single-cell profiling^27^. The 2-step process included STA (specific target amplification) reaction (RT-STA cycling conditions and an Exonuclease I treatment method to remove unincorporated primers). The RT-STA solution (9.0 μl per well) consisted of: 5 μl CellsDirect 2X Reaction Mix/0.2 μl SuperScript III RT Platinum Taq Mix/1 μl 10X Primer Mix (500 μM)/2.8 μl Nuclease Free H2O (Invitrogen). The thermal cycling profile protocol was as follows: Stage 1: 50 °C, 15 min; Stage 2: 95 °C, 2 min; Stage 3: 20 cycles of (95 °C, 15 sec; and 60 °C, 4 min). The Exo I treatment method (total volume 3.5 μl [per 9 μl RT-STA solution]) consisted of: 2.52 μl Nuclease Free H2O, 0.36 μl Exonuclease I Reaction Buffer (10X), and 0.72 μl Exonuclease I (20units/μl) (New England BioLabs). The thermal cycling protocol was as follows: Stage 1: 37 °C, 30 min; Stage 2: 80 °C, 15 min. The final concentration of STAreaction + Exonuclease I was diluted 5-fold in this experiment. The human cDNA library (Biochain) was treated with RTA-STA/ExoI treatment method, diluted 5-fold with additional serial dilutions (1:3). In the NTC, negative control condition, no cells were sorted at all but RT-STA was applied. 5 primer assays were used (SABiosciences) with GAPDH as housekeeping gene and 4 high expressing genes reflecting involvement of multiple pathways (EGF/ENG/NOTCH/TIMP1).

## Tumorsphere Analysis

### HemSC-derived Tumorspheres

The *in vitro* tumorsphere formation assay involved a three-dimensional (3D) culture system with two main approaches. The first approach for tumorsphere growth consisted in the growth of spheres as a suspension in non-adherent 6-well plates, and the second approach involved the culture of spheres on Matrigel- or fibronectin-coated plates (BD Biosciences). STEMPro hESC- human embryonic stem cell culture medium (Invitrogen) or EBM-2 medium supplemented with 20% FBS and penicillin/streptomycin (Lonza) was used.

In one week of culture in non-adhesive plates, the cells in suspension started to form tumorspheres that grew in suspension or loosely attached to the culture substrate. The tumorspheres were captured with a sterile 25-ml serological pipet for analysis (by cytospin or paraffin embedded plasma-thrombin clot formation as described). For *in vitro* differentiation analysis, the spheres were transferred on Matrigel- or fibronectin-coated plates (BD Biosciences).

For immunohistochemical analysis, the culture medium was gently removed from the culture dishes. Human plasma was added, followed by the addition of thrombin. The spheres remained embedded in the plasma-thrombin clot were paraffin-embedded. Sections of paraffin-embedded plasma-thrombin clots were stained with hematoxylin and eosin (H&E) and for validation studies, tumorsphere sections were stained with 1) GLUT1 antibody – diagnostic clinical marker for IH; and 2) CD44 highest expressing marker in heterogeneous cell surface marker screening panel. For independent validation, the NYU Immunohistochemistry (IHC) Core Facility processed the sections according to the manufacturers’ protocols. The primary antibodies used for immunohistochemical analysis (provided by Dr. Luis Chiriboga, NYU IHC Core Facility) were GLUT1 (Ventana 760-4526) and CD44 (Dako M7082). The primary antibody was detected with biotinylated goat anti mouse IgG antibodies (provided in the detection kit from the respective manufacturers). Additional controls included the use of tissue microarray for IHC.

For tumorsphere HT bioimaging and differentiation analysis, the HemSC-derived tumorspheres and differentiated derivatives were stained for the pericyte cell surface marker PDGFR-β (purified antibody to CD140b [clone 28D4]) (BD Biosciences) and directly conjugated with AlexaFluor 647 goat anti-mouse Ig. Prior to live imaging, tumorspheres and derivatives were stained with cell-permeable nucleic acid stain - Hoechst 33342 (BD Pharmingen).

## Additional Information

**How to cite this article**: Harbi, S. *et al*. Infantile Hemangioma Originates From A Dysregulated But Not Fully Transformed Multipotent Stem Cell. *Sci. Rep.* **6**, 35811; doi: 10.1038/srep35811 (2016).

**Publisher’s note:** Springer Nature remains neutral with regard to jurisdictional claims in published maps and institutional affiliations.

## Supplementary Material

Supplementary Information

Supplementary Video 1

Supplementary Video 2

Supplementary Video 3

## Figures and Tables

**Figure 1:**
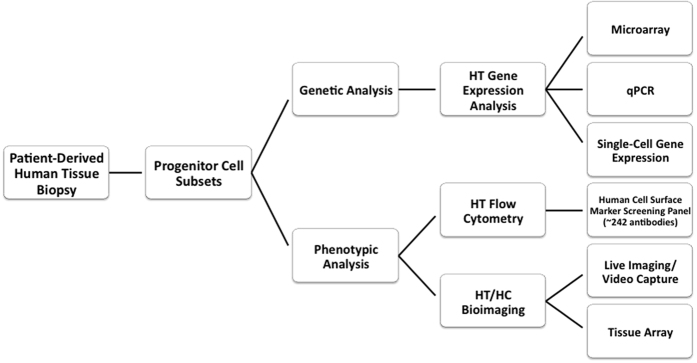
Research Workflow. To analyze the HemSC and Hem-derivative cells involved in IH pathogenesis we used cellular, molecular, and genetic techniques. We used flow cytometry (to complement the gene expression analysis) for assay development of a comprehensive panel of potential biomarkers and therapeutic targets. Single-cell analysis of progenitor cell subsets included profiling of mRNA transcripts and proteins.

**Table 1:** Microarray analysis of angiogenesis signaling.

ProbeSet Details	Gene	ANOVA (Fold-Change)
ref|Homo sapiens chemokine (C-X-C motif) ligand 5 (CXCL5), mRNA **[NM_002994]**	CXCL5**	1415.37
ref|Homo sapiens neuropilin 1 (NRP1), transcript variant 1, mRNA **[NM_003873]**	NRP1**	359.955
ref|Homo sapiens hepatocyte growth factor (hepapoietin A; scatter factor) (HGF), transcript variant 2, mRNA **[NM_001010931]**	HGF*	232.701
ref|Homo sapiens vascular endothelial growth factor B (VEGFB), transcript variant VEGFB-186, mRNA **[NM_003377]**	VEGFB	127.29
ref|Homo sapiens plasminogen activator, urokinase (PLAU), transcript variant 2, mRNA **[NM_001145031]**	PLAU	124.794
ref|Homo sapiens inhibitor of DNA binding 1, dominant negative helix-loop-helix protein (ID1), transcript variant 1, mRNA **[NM_002165]**	ID1*	103.306
ref|Homo sapiens vascular endothelial growth factor A (VEGFA), transcript variant 6, mRNA **[NM_001025370]**	VEGFA	71.426
ref|Homo sapiens interleukin 8 (IL8), mRNA **[NM_000584]**	IL8**	61.9279
ref|Homo sapiens hypoxia inducible factor 1, alpha subunit (basic helix-loop-helix transcription factor) (HIF1A), transcript variant 2, mRNA **[NM_181054]**	HIF1A	29.8251
ref|Homo sapiens alanyl (membrane) aminopeptidase (ANPEP), mRNA **[NM_001150]**	ANPEP**	25.4382
ref|Homo sapiens vascular endothelial growth factor C (VEGFC), mRNA **[NM_005429]**	VEGFC**	6.86366
ref|Homo sapiens neuropilin 2 (NRP2), transcript variant 1, mRNA **[NM_201266]**	NRP2	5.57189

^*^HemSC GLUT1+ Subset P-Value (unadjusted p-value) <0.05.

^**^HemSC GLUT1+ Subset P-Value (FDR) <0.05.

**Table 2:** qPCR analysis of angiogenesis signaling.

Unigene	GeneBank	Symbol	Description	Gene Name	AVG C_T_ HUVEC	AVG C_T_ HemSC	Standard Deviation HemSC	Fold Change
Hs.1239	NM_001150	ANPEP	Alanyl (membrane) aminopeptidase	APN, CD13, GP150, LAP1, P150, PEPN	19.67	13.78	0.734533	16.3506
Hs.89714	NM_002994	CXCL5	Chemokine (C-X-C motif) ligand 5	ENA-78, SCYB5	29.2	15.75	0.698162	3099.1871
Hs.396530	NM_000601	HGF	Hepatocyte growth factor (hepapoietin A; scatter factor)	DFNB39, F-TCF, HGFB, HPTA, SF	32.62	20.04	0.456547	1694.144
Hs.597216	NM_001530	HIF1A	Hypoxia inducible factor 1, alpha subunit (basic helix-loop-helix transcription factor)	HIF-1alpha, HIF1, HIF1-ALPHA, MOP1, PASD8, bHLHe78	22.77	15.51	0.780008	42.422
Hs.504609	NM_002165	ID1	Inhibitor of DNA binding 1, dominant negative helix-loop-helix protein	ID, bHLHb24	28.63	17.04	1.647472	852.6683
Hs.624	NM_000584	IL8	Interleukin 8	CXCL8, GCP-1, GCP1, LECT, LUCT, LYNAP, MDNCF, MONAP, NAF, NAP-1, NAP1	25.57	15.84	1.773494	235.4604
Hs.131704	NM_003873	NRP1	Neuropilin 1	BDCA4, CD304, DKFZp686A03134, DKFZp781F1414, NP1, NRP, VEGF165R	24.83	16.31	1.31957	100.9157
Hs.471200	NM_003872	NRP2	Neuropilin 2	MGC126574, NP2, NPN2, PRO2714, VEGF165R2	34.36	21.92	2.198745	1533.353
Hs.77274	NM_002658	PLAU	Plasminogen activator, urokinase	ATF, UPA, URK, u-PA	31.59	19.74	2.04359	1013.6478
Hs.73793	NM_003376	VEGFA	Vascular endothelial growth factor A	MGC70609, MVCD1, VEGF, VPF	29.44	21.4	0.324989	72.8576
Hs.78781	NM_003377	VEGFB	Vascular endothelial growth factor B	VEGFL, VRF	26.54	19.43	1.735138	38.2081
Hs.435215	NM_005429	VEGFC	Vascular endothelial growth factor C	Flt4-L, VRP	22	16.99	1.636962	8.9301

**Table 3:** Microarray analysis of cell surface markers.

ProbeSet Details	Gene	CD Nomenclature	ANOVA (Fold-Change)
ref|Homo sapiens 5′-nucleotidase, ecto (CD73) (NT5E), transcript variant 1, mRNA **[NM_002526]**	NT5E**	CD73	261.611
ref|Homo sapiens interleukin 1 receptor, type I (IL1R1), mRNA **[NM_000877]**	IL1R1**	CD121a	257.625
ref|Homo sapiens solute carrier family 7 (amino acid transporter light chain, L system), member 5 (SLC7A5), mRNA **[NM_003486]**	SLC7A5**	CD98	231.093
ref|Homo sapiens Thy-1 cell surface antigen (THY1), mRNA **[NM_006288]**	THY1*	CD90	153.582
ref|Homo sapiens 5′-nucleotidase, ecto (CD73) (NT5E), transcript variant 1, mRNA **[NM_002526]**	NT5E**	CD73	152.496
ref|Homo sapiens CD44 molecule (Indian blood group) (CD44), transcript variant 1, mRNA **[NM_000610]**	CD44**	CD44	110.552
ref|Homo sapiens membrane metallo-endopeptidase (MME), transcript variant 2b, mRNA **[NM_007289]**	MME**	CD10	98.0114
ref|Homo sapiens interleukin 4 receptor (IL4R), transcript variant 2, mRNA **[NM_001008699]**	IL4R**	CD124	90.9778
ref|Homo sapiens CD55 molecule, decay accelerating factor for complement (Cromer blood group) (CD55), transcript variant 1, mRNA **[NM_000574]**	CD55*	CD55	74.8856
ref|Homo sapiens epidermal growth factor receptor (EGFR), transcript variant 1, mRNA **[NM_005228]**	EGFR*	EGF Receptor	70.8004
ref|Homo sapiens low density lipoprotein receptor-related protein 1 (LRP1), mRNA **[NM_002332]**	LRP1**	CD91	68.725
ref|Homo sapiens CD47 molecule (CD47), transcript variant 2, mRNA **[NM_198793]**	CD47*	CD47	49.6924
ref|Homo sapiens low density lipoprotein receptor-related protein 1 (LRP1), mRNA **[NM_002332]**	LRP1*	CD91	40.2019
ref|Homo sapiens poliovirus receptor-related 2 (herpesvirus entry mediator B) (PVRL2), transcript variant alpha, mRNA [NM_002856]	PVRL2	CD112	29.085
ref|Homo sapiens alanyl (membrane) aminopeptidase (ANPEP), mRNA **[NM_001150]**	ANPEP**	CD13	25.4382
ref|Homo sapiens solute carrier family 2 (facilitated glucose transporter), member 1 (SLC2A1), mRNA **[NM_006516]**	SLC2A1**	GLUT1	25.4269
ref|Homo sapiens platelet-derived growth factor receptor, beta polypeptide (PDGFRB), mRNA **[NM_002609]**	PDGFRB	CD140b	24.9491
ref|Homo sapiens integrin, alpha 2 (CD49B, alpha 2 subunit of VLA-2 receptor) (ITGA2), mRNA **[NM_002203]**	ITGA2*	CD49b	23.3088
ref|Homo sapiens interleukin 3 receptor, alpha (low affinity) (IL3RA), mRNA **[NM_002183]**	IL3RA*	CD123	17.3501
ref|Homo sapiens integrin, alpha 3 (antigen CD49C, alpha 3 subunit of VLA-3 receptor) (ITGA3), transcript variant a, mRNA **[NM_002204]**	ITGA3**	CD49c	16.6081
ref|Homo sapiens Fas (TNF receptor superfamily, member 6) (FAS), transcript variant 1, mRNA **[NM_000043]**	FAS	CD95	16.3111
ref|Homo sapiens CD59 molecule, complement regulatory protein (CD59), transcript variant 1, mRNA **[NM_203330]**	CD59*	CD59	15.1882
ref|Homo sapiens interleukin 10 receptor, beta (IL10RB), mRNA **[NM_000628]**	IL10RB	CD210	14.3943
ref|Homo sapiens Fas (TNF receptor superfamily, member 6) (FAS), transcript variant 1, mRNA **[NM_000043]**	FAS	CD95	14.3102
ref|Homo sapiens platelet-derived growth factor receptor, alpha polypeptide (PDGFRA), mRNA **[NM_006206]**	PDGFRA	CD140a	13.6688
ref|Homo sapiens integrin, alpha 4 (antigen CD49D, alpha 4 subunit of VLA-4 receptor) (ITGA4), mRNA **[NM_000885]**	ITGA4	CD49d	11.8331
ref|Homo sapiens major histocompatibility complex, class II, DP beta 1 (HLA-DPB1), mRNA **[NM_002121]**	HLA-DPB1	HLA-DR, DP, DQ	11.7186
ref|Homo sapiens plasminogen activator, urokinase receptor (PLAUR), transcript variant 3, mRNA **[NM_001005377]**	PLAUR	CD87	11.5119
ref|Homo sapiens CD27 molecule (CD27), mRNA **[NM_001242]**	CD27	CD27	10.4003
ref|Homo sapiens interleukin 4 receptor (IL4R), transcript variant 1, mRNA **[NM_000418]**	IL4R*	CD124	9.98291

^*^HemSC GLUT1+ Subset P-Value (unadjusted p-value) <0.05

^**^HemSC GLUT1+ Subset P-Value (FDR) <0.05.

**Table 4:** Analysis of cell surface markers.

**AP:**	Mulliken and Glowacki biologic classification of **differentiating hemangiomas** benchmark **pluripotent stem cell marker**
**CD10:**	(CALLA; Neprilysin; membrane metalloendopeptidase) **promotes endothelial cell growth** and **angiogenesis** in the tumor microenvironment
**CD13:**	(metalloprotease aminopeptidase N; ANPEP/APN) potent regulator in **angiogenesis** and is overexpressed in tumor cells
**CD44:**	key **CSC marker** in multiple types of tumors a high expressing marker in IH
**CD47:**	a “**don’t eat me**” signal for phagocytic cells expressed on the surface of all human solid tumor cells overexpressed on cancer stem cells to **evade immunosurveillance**
**CD59/CD46:**	**prevents** complement mediated **tumor cell lysis** Protectin or CD59 – powerful inhibitor of complement cytolysis CD46, a complement regulatory protein, inhibits complement activation and serves to protect the host cell against attack
**CD73:**	generation of adenosine **suppresses T-cell response** is involved in both tumorigenic and metastatic potential
**CD105:**	(Endoglin) of TGF-β pathway expressed in the endothelial cells (**angiogenesis**) of glioblastoma (GBM)
**CD147:**	(extracellular matrix metalloproteinase inducer **EMMPRIN**; Neurotelin; Basigin/BSG) increased expression in tumors and in fetal development

Cell Surface Markers Include: Alkaline Phosphatase (AP), CD10, CD13, CD44, CD46, CD47, CD59, CD73, CD105, CD147.

**Table 5:** Flow cytometry analysis of cell surface markers.

Signature Markers^a^:	Glioblastoma^b^	IH^c^
Disialoganglioside GD2	**+++**	
Vβ23	**+++**	
CD59	**++**	**+++**
CD44	**+**	**+++**
CD151	**+/++**	**+/++**
CD73	**+**	**++**
CD147	**++**	**++**
CD13	****	**++**

^**a**^Represents a sample set (~240 monoclonal cell surface markers).

^**b**^Glioblastoma tumor sphere formation - a dysregulated, fully transformed cancer stem cell model.

^**c**^IH tumor sphere formation - a dysregulated, but ***not*** fully transformed stem cell model. AF647 % Positive, +, 0.1–20; ++, 21–80; +++, 81–100.

**Table 6:** Panel of IH biomarkers for multiparameter analysis and assay development for clinical research.

Gene	CD/Nomenclature
ANPEP**	CD13
BSG	CD147
CD151	CD151
CD27	CD27
CD44**	CD44
CD47	CD47
CD55	CD55
CD59	CD59
CD81	CD81
CXCL5	CXCL5
EGFR	EGF Receptor
ENG	CD105
HGF	HGF
ID1	ID1
IL1R1	CD121a
IL4R	CD124
IL8	CXCL8
ITGA2	CD49b
ITGA3	CD49c
ITGA4	CD49d
KLF10	KLF10
LEPR	CD295
LRP1	CD91
MME**	CD10
NRP1	CD304
NRP2	NRP2
NT5E**	CD73
PDGFRA	CD140a
PDGFRB	CD140b
PPARD	PPARD
PPARG	PPARG
PROM 1/2	CD133
PTGS1	PTGS1
RXRB	RXRB
SALL4	SALL4
SLC2A1*	GLUT1
SP1	SP1
THY1	CD90
TRAV24 (T cell receptor alpha variable 24)	Invariant NKT
VEZF1	VEZF1

*IH Clinical Marker.

**IH Signature Cell Surface Markers - for *in vitro* diagnostic assays (such as IHC).
