# Supplementary material for: Infantile Hemangioma Originates From A Dysregulated But Not Fully Transformed Multipotent Stem Cell
Source: Sci Rep. 2016 Oct 27;6:35811. doi: 10.1038/srep35811 (PMC5081534; doi:10.1038/srep35811)
Supplement: Supplementary Information [file srep35811-s1.pdf]

INFANTILE HEMANGIOMA ORIGINATES FROM A DYSREGULATED BUT NOT FULLY TRANSFORMED MULTIPOTENT STEM CELL

Shaghayegh Harbi[1]*, Rong Wang[2], Michael Gregory[3], Nicole Hanson[3], Keith Kobylarz[3], Kamilah Ryan[3], Yan Deng[3], Peter Lopez[3], Luis Chiriboga[3] and Paolo Mignatti[1, 4]

Department of [1]Medicine, New York University School of Medicine, 550 First Avenue. New York, NY 10016, USA

Department of [2]Neurosurgery, Memorial Sloan Kettering Cancer Center, New York, New York 10065, USA

Departments of [3]Pathology and [4]Cell Biology, New York University School of Medicine, 550 First Avenue. New York, NY 10016, USA

Corresponding Author: Shaghayegh Harbi (harbi@nyu.edu)

- **Supplementary Figure 1. Gene expression analysis of angiogenesis signaling. a. qPCR analysis.** 96-well qPCR arrays (SABiosciences) were used to examine expression profiles of genes in sorted cell populations. Fold change/regulation was calculated using delta delta C_t method. The values were compared to fold change gene expression analysis using one-way analysis of variance (ANOVA) of samples (Partek Genomics Suite software). **b. Microarray and qPCR analysis of gene expression in HemSC relative to HUVEC.** The figure represents fold change calculations using ANOVA for qPCR and microarray gene expression analysis to compare the normalized expression of significant genes in the angiogenesis signaling gene panel between HemSC $^{GLUT1+}$ and HUVEC (control).

- **Supplementary Figure 2. Microarray analysis of drug targeting pathways**. Agilent whole genome oligo microarrays (one-color – Cy3 labeling) were used to examine expression profiles of genes in druggable (mTOR and Adipocytokine/PPAR Signaling Pathway). Microarray gene expression analysis was performed using one-way analysis of variance (ANOVA) of samples (Partek Genomics Suite software) to compare the normalized expression of significant genes between HemSC $^{GLUT1+}$ and HUVEC (control). The dot plots compare the expression of significant genes between HemSC $^{GLUT1+}$ and HUVEC (control) to exhibit large gene expression changes (HemSC $^{GLUT1+}$ - **H**, HUVEC endothelial progenitor (control) - **E**, Glioblastoma CSC – **G**). The gene expression intensity data images were generated with Partek Genomics Suite Software. **a. mTOR microarray gene significance dot plot.** The gene expression of mTOR (mammalian target of rapamycin), a drug-target, showed high variability. However, Protein Kinase B (AKT1), a kinase upstream of mTOR, was consistently over-expressed. **b. Microarray analysis of the PPAR/Adipocytokine signaling pathway.** The PPAR/ Adipocytokine signaling pathway microarray analysis showed significant over-expression of transcription factors (PPARD, PPARG, KLF10, RXRB) and the clinical biomarker of IH, GLUT1 (SLC2A1). Representative dot plots and fold-change bar graph are shown.

- **Supplementary Figure 3. HemSC-derived tumorsphere paraffin embedded plasma-thrombin clots.** Tumorsphere formation in culture wells was characterized by immunohistochemical analysis of paraffin embedded plasma-thrombin clots including the tumorspheres as described in Methods.

- **Supplementary Figure 4. Immunohistochemical analysis of human tissues.** CD44 is highly expressed in IH cells, as shown by our genotypic and phenotypic analyses, and can be detected by immunohistochemistry in various normal and neoplastic tissues by analyzing a comprehensive human tissue array, which includes biological replicates.

SUPPLEMENTARY FIGURE 1a

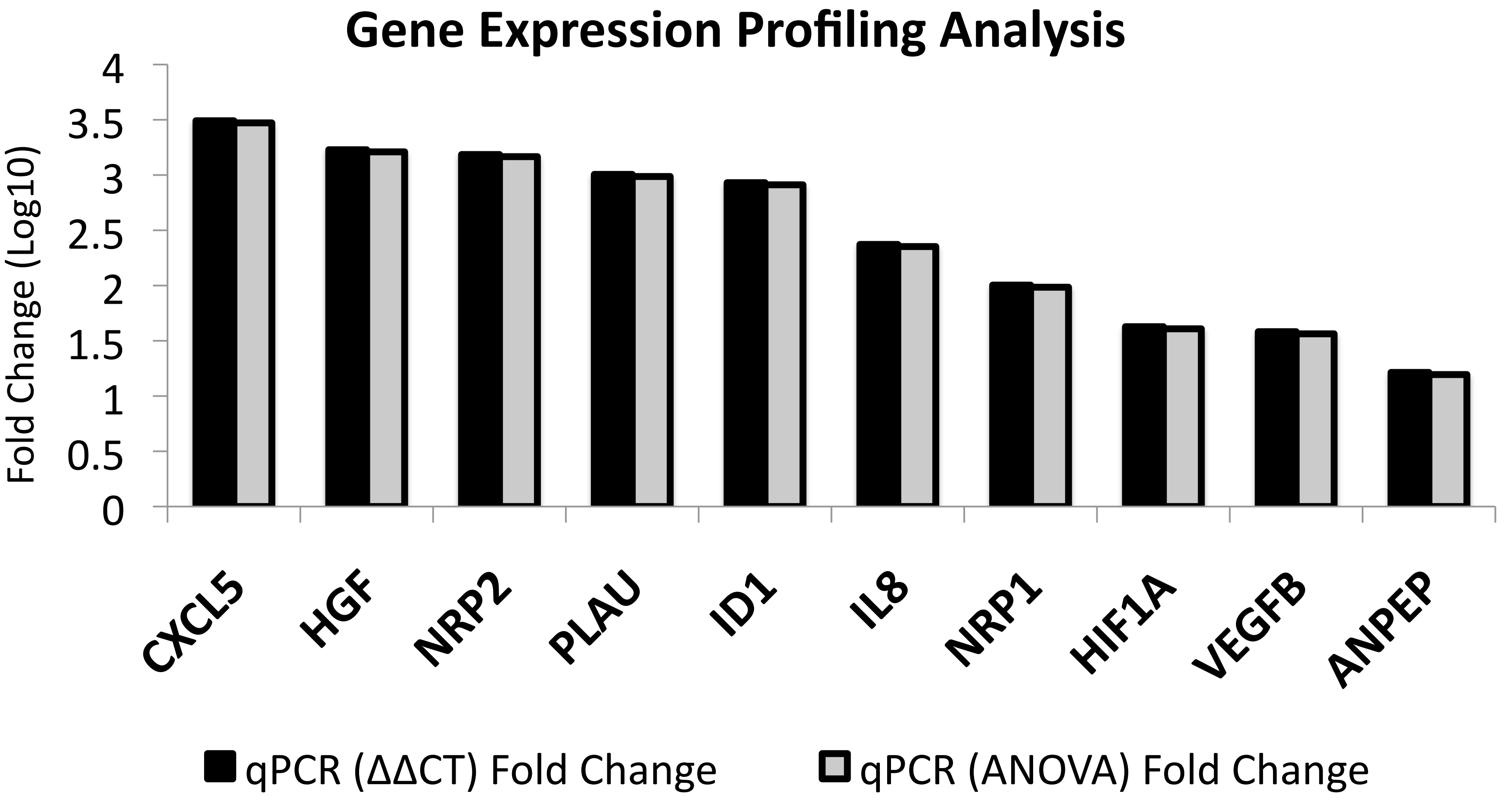

SUPPLEMENTARY FIGURE 1b

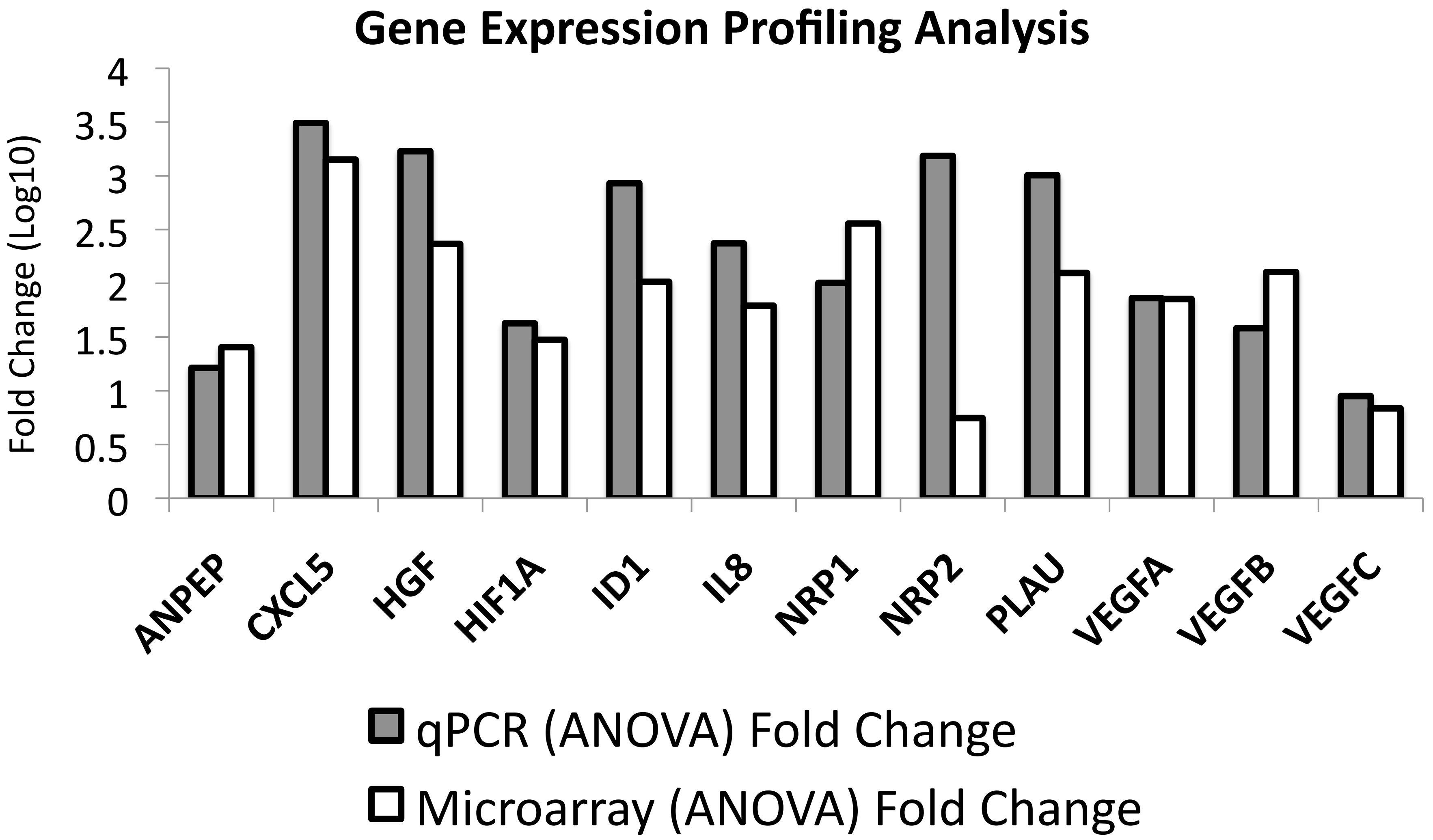

SUPPLEMENTARY FIGURE 2a

mTOR Signaling
(mammalian target of rapamycin)

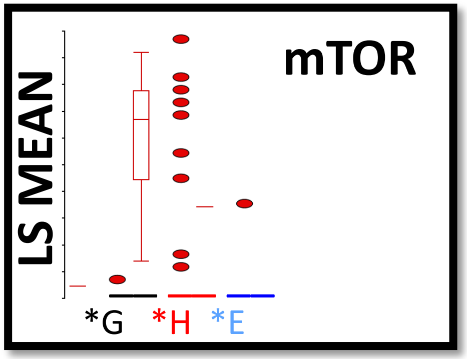

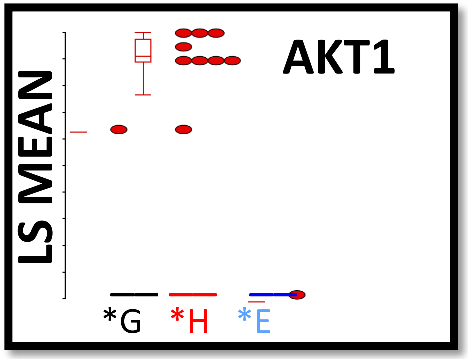

SUPPLEMENTARY FIGURE 2b

Adipocytokine & PPAR Signaling Pathway

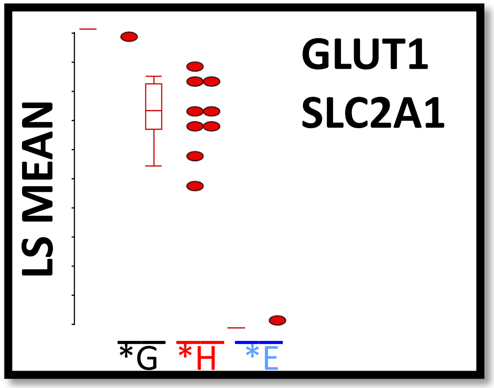

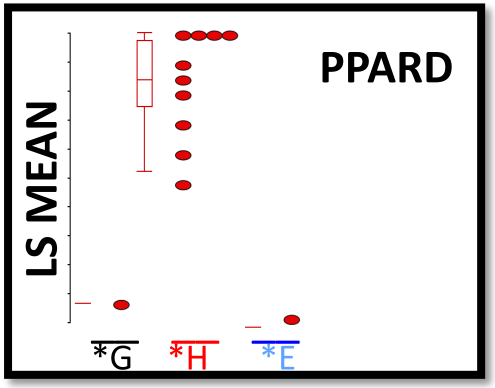

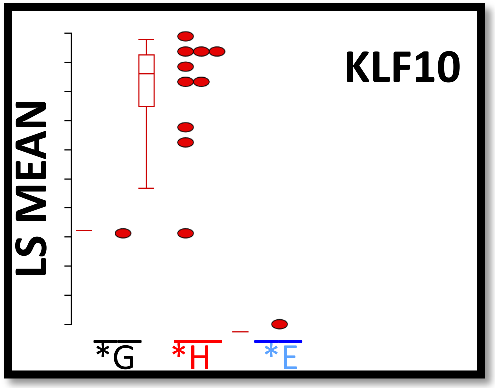

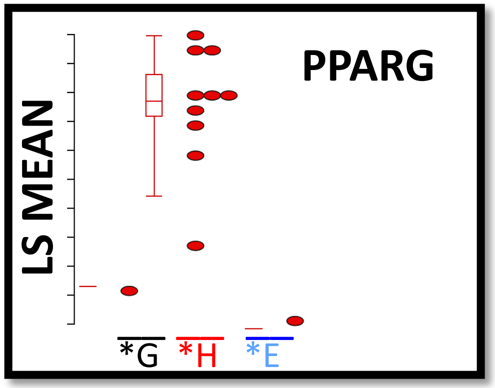

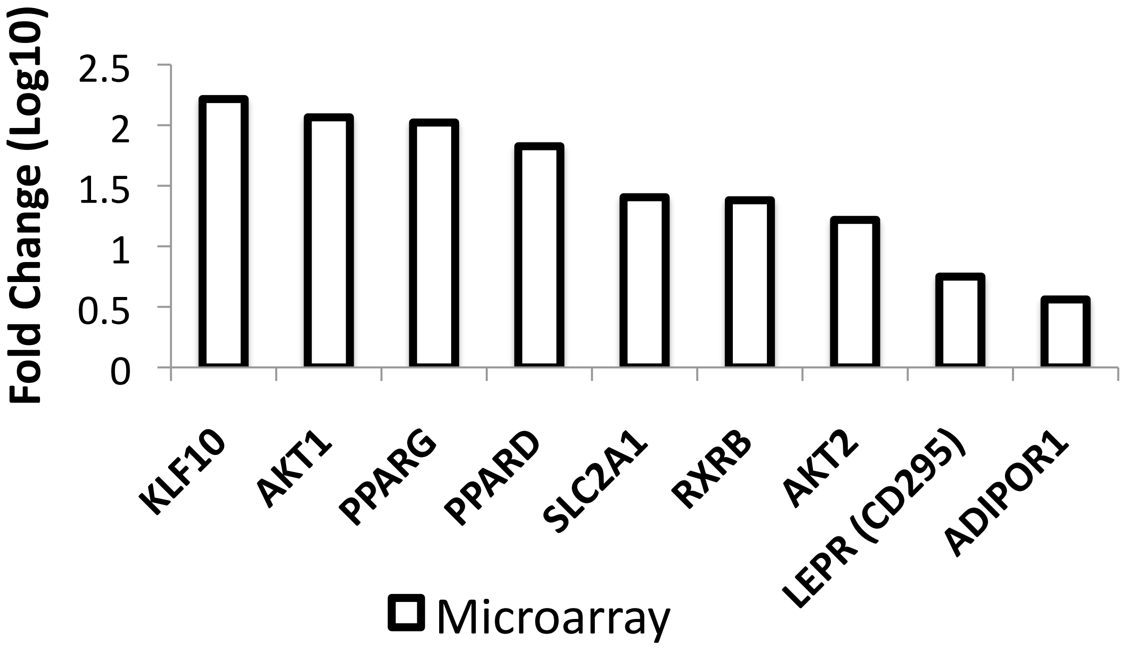

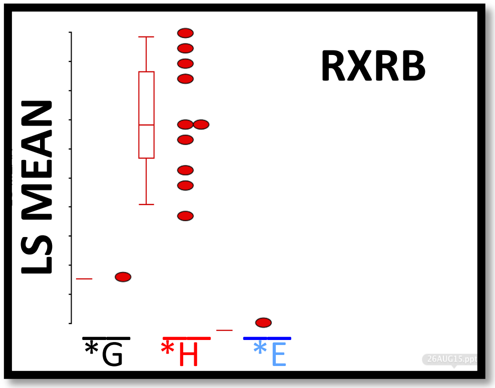

Transcription Factors: PPARD, PPARG, KLF10, RXRB

IH Clinical Biomarker: GLUT1

SUPPLEMENTARY FIGURE 3

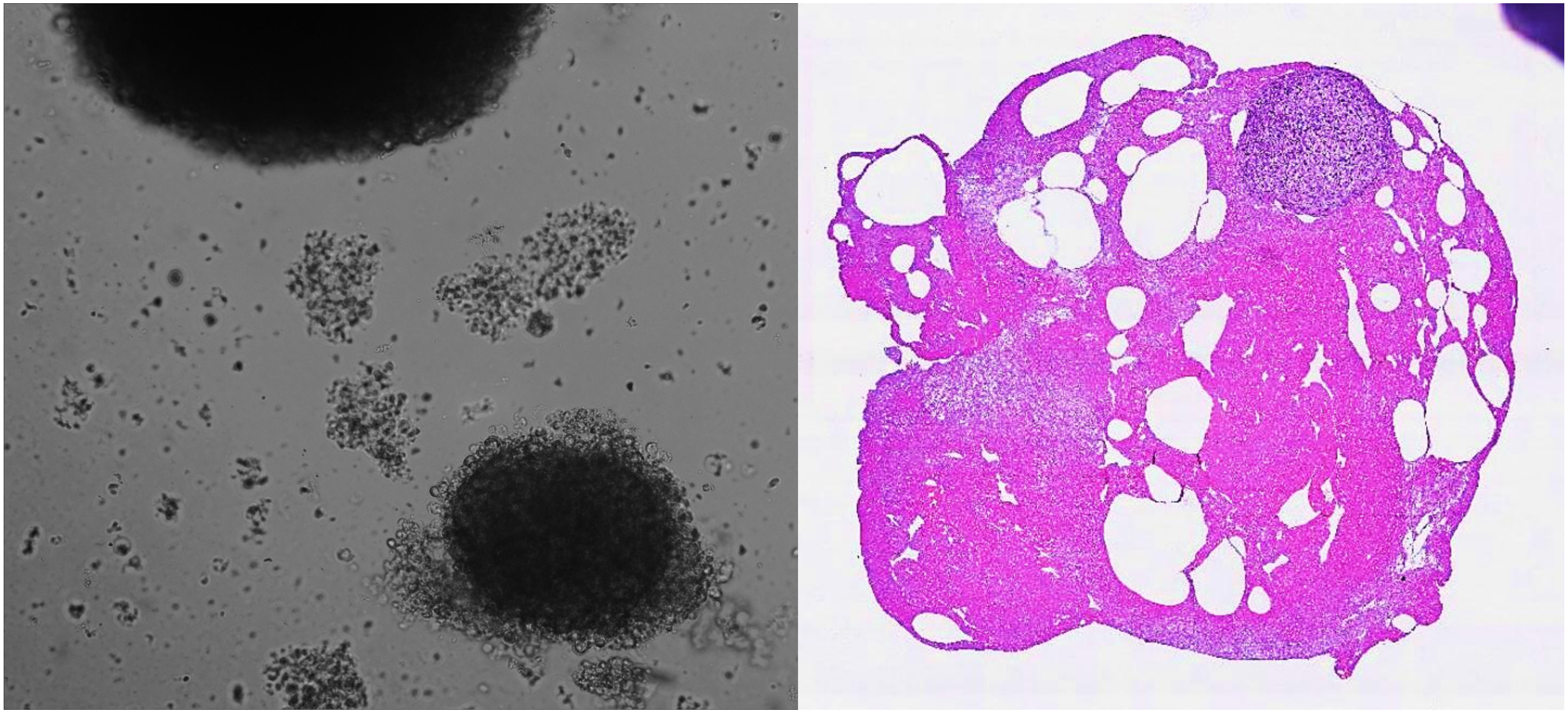

SUPPLEMENTARY FIGURE 4a

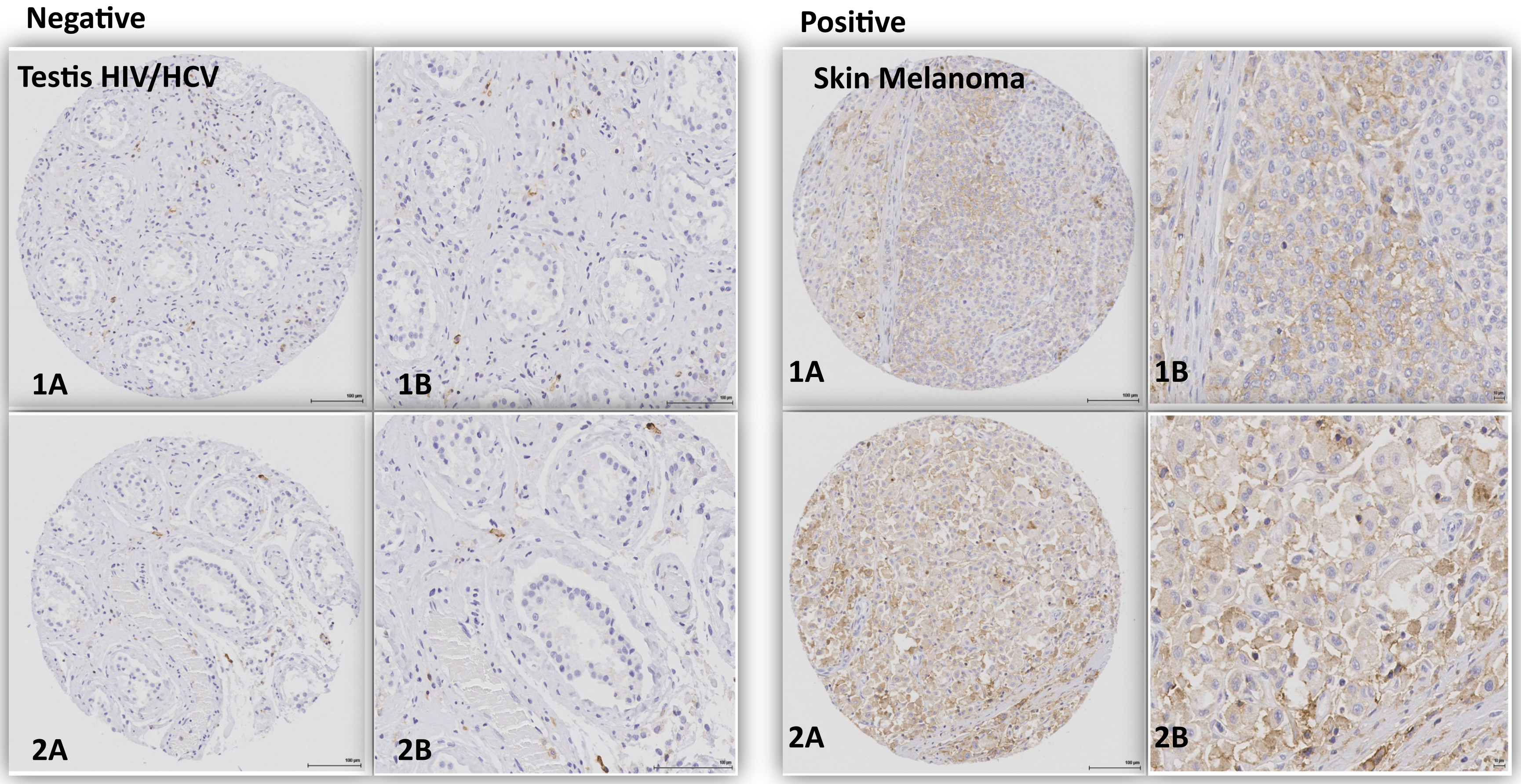

SUPPLEMENTARY FIGURE 4b

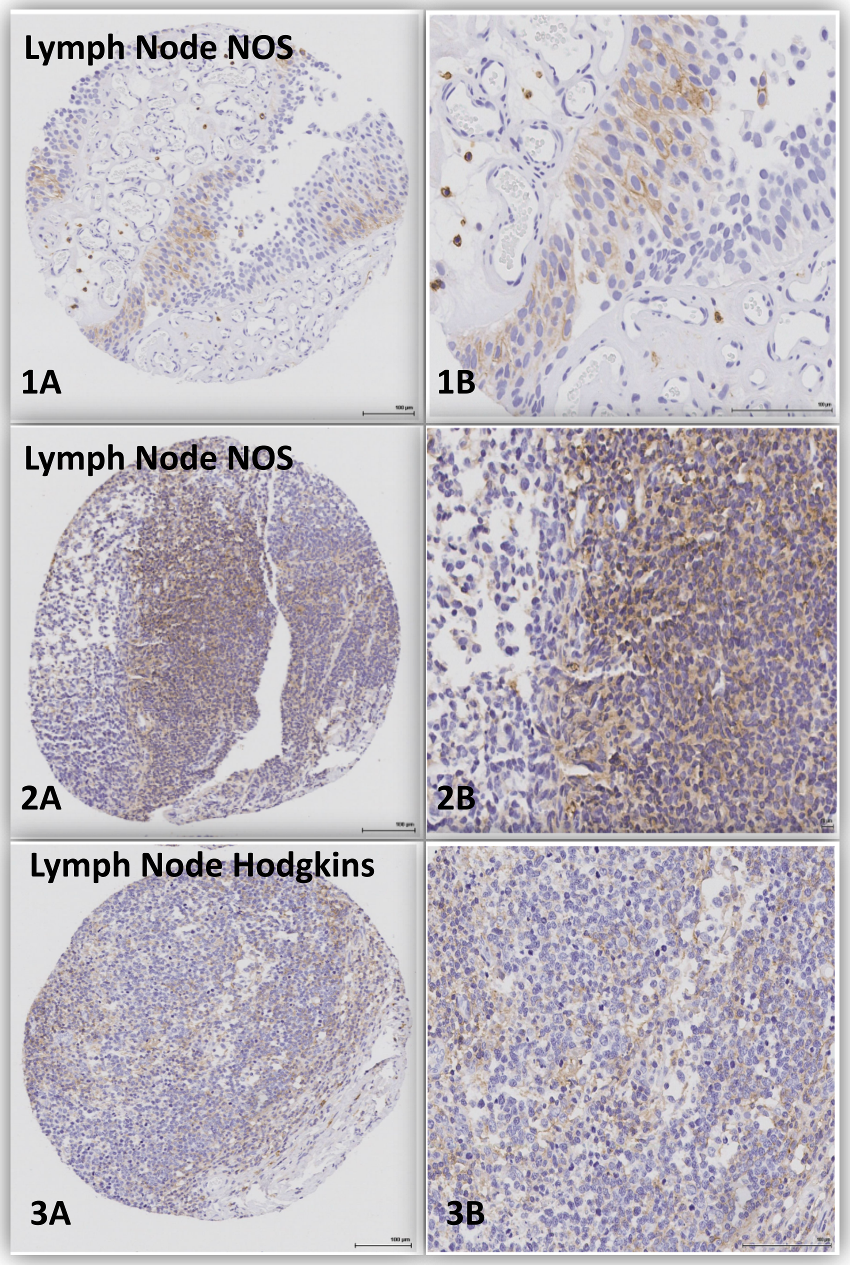

- **Supplementary Video 1-3. HemPericyte derivative bioimaging analysis shows *de novo* formation of derivatives from undifferentiated HemSCs.** The differentiated derivatives from the tumorspheres were characterized by immunostaining for the pericyte surface marker PDGFR-β. Using the Applied Precision Personal DV live-cell imaging system, the study design included 21-h imaging studies with live imaging of HemSC-derived tumorsphere and derivative formation/interaction (Video 1-3).
